# Applications of VirScan to broad serological profiling of bat reservoirs for emerging zoonoses

**DOI:** 10.3389/fpubh.2023.1212018

**Published:** 2023-09-22

**Authors:** Emily Cornelius Ruhs, Wan Ni Chia, Randy Foo, Alison J. Peel, Yimei Li, H. Benjamin Larman, Aaron T. Irving, Linfa Wang, Cara E. Brook

**Affiliations:** ^1^Department of Ecology and Evolution, University of Chicago, Chicago, IL, United States; ^2^Grainger Bioinformatics Center, Field Museum of Natural History, Chicago, IL, United States; ^3^Program in Emerging Infectious Diseases, Duke-NUS Medical School, Singapore, Singapore; ^4^CoV Biotechnology Pte Ltd., Singapore, Singapore; ^5^Centre for Planetary Health and Food Security, School of Environment and Science, Griffith University, Brisband, QLD, Australia; ^6^Quantitative and Computational Biology, Princeton University, Princeton, NJ, United States; ^7^HBL – Institute for Cell Engineering, Division of Immunology, Department of Pathology, Johns Hopkins University, Baltimore, MD, United States; ^8^Second Affiliated Hospital of Zhejiang University, Zhejiang University School of Medicine, Hangzhou, Zhejiang, China; ^9^Zhejiang University-University of Edinburgh Institute, Haining, Zhejiang, China; ^10^BIMET - Biomedical and Translational Research Centre of Zhejiang Province, Zhejiang Province, China; ^11^SingHealth Duke-NUS Global Health Institute, Singapore, Singapore

**Keywords:** Chiroptera, PhIP-Seq, surveillance, VirScan, virus

## Abstract

**Introduction:**

Bats are important providers of ecosystem services such as pollination, seed dispersal, and insect control but also act as natural reservoirs for virulent zoonotic viruses. Bats host multiple viruses that cause life-threatening pathology in other animals and humans but, themselves, experience limited pathological disease from infection. Despite bats’ importance as reservoirs for several zoonotic viruses, we know little about the broader viral diversity that they host. Bat virus surveillance efforts are challenged by difficulties of field capture and the limited scope of targeted PCR- or ELISA-based molecular and serological detection. Additionally, virus shedding is often transient, thus also limiting insights gained from nucleic acid testing of field specimens. Phage ImmunoPrecipitation Sequencing (PhIP-Seq), a broad serological tool used previously to comprehensively profile viral exposure history in humans, offers an exciting prospect for viral surveillance efforts in wildlife, including bats.

**Methods:**

Here, for the first time, we apply PhIP-Seq technology to bat serum, using a viral peptide library originally designed to simultaneously assay exposures to the entire human virome.

**Results:**

Using VirScan, we identified past exposures to 57 viral genera—including betacoronaviruses, henipaviruses, lyssaviruses, and filoviruses—in semi-captive *Pteropus alecto* and to nine viral genera in captive *Eonycteris spelaea*. Consistent with results from humans, we find that both total peptide hits (the number of enriched viral peptides in our library) and the corresponding number of inferred past virus exposures in bat hosts were correlated with poor bat body condition scores and increased with age. High and low body condition scores were associated with either seropositive or seronegative status for different viruses, though in general, virus-specific age-seroprevalence curves defied assumptions of lifelong immunizing infection, suggesting that many bat viruses may circulate via complex transmission dynamics.

**Discussion:**

Overall, our work emphasizes the utility of applying biomedical tools, like PhIP-Seq, first developed for humans to viral surveillance efforts in wildlife, while highlighting opportunities for taxon-specific improvements.

## Introduction

Zoonotic viruses with bats as the natural reservoir host cause higher case fatality rates in humans than do viruses derived from any other mammalian or avian host (1–3). Among these viruses, bats are confirmed reservoir hosts for Hendra and Nipah henipaviruses (4, 5), Marburg filovirus (6), rabies lyssavirus (7), and many coronaviruses (8, 9). These associations highlight the great public health importance of continued surveillance for and discovery of novel bat-hosted viruses.

While a wide variety of known zoonotic viruses originate in bats, bats themselves do not appear to experience substantial clinical disease from these infections (10), except rabies and related lyssaviruses, and potentially, Lloviu filovirus and Tacaribe arenavirus (11–15). It is hypothesized that bats rely on several unique innate and cell-mediated immune mechanisms for virus control (16), including constitutive expression of the antiviral cytokine, interferon (IFN), in certain species (17). Bats are also known to mount measurable antibody responses after infection—which continue to be produced by long-term memory B cells—but the functional role of antibodies in bat anti-viral responses and the durability of those responses are still being elucidated (4, 18–21). For example, experimental infection of bats with Marburg virus, Ebolavirus, and Sosuga virus have produced varying results on the ability of antibodies to neutralize viral infection and the duration of that protection (4, 21). Nonetheless, *Rousettus aegyptiacus* bats previously infected with Marburg virus did mount rapid antibody responses after reinfection (19, 22, 23). Jamaican fruit bats (*Artibeus jamaicensis*) experimentally infected with Tacaribe virus showed upregulation of innate antiviral responses, but also elevated immunoglobulin expression in certain tissues (24). Antibody-mediated immunity generates specific responses to specific viruses that can provide long-lasting protection for many years (25, 26). As a result, serological surveys offer a powerful tool to elucidate current and past viral exposures.

Previous serological studies in bats have relied primarily on ELISA, virus neutralization, or Luminex technology (9, 27–29), which offer powerful insights into the landscape of past exposure for a specific subset of targeted antigens. Nonetheless, these technologies are constrained in the breadth of potential targets that can be simultaneously assayed (thus limiting their utility in truly exploratory surveillance settings) and/or necessitate technically difficult protein synthesis and purification (30, 31).

Recent technological advancements in the biomedical field could be applied to wildlife systems to expand the breadth of serological surveillance. Phage ImmunoPrecipitation Sequencing (PhIP-Seq) combines high-throughput oligonucleotide library synthesis (OLS) and next generation DNA sequencing technology to identify antibody-specific binding to peptides displayed on bacteriophages, which collectively comprise a target “peptidome” that can be proteome-wide in scale (32, 33). PhIP-Seq technology has previously been used to comprehensively profile human serum samples for antibodies to the “VirScan” peptidome, a peptide library representing over 200 viruses (9,449 proteins) that constitute the complete known human virome (32). The high throughput structure of PhIP-Seq offers considerable advantages over ELISA-based technologies that demand a different, targeted assay be carried out for each virus under investigation (34). Additionally, the simple *in silico* design and commercial synthesis of a PhIP-Seq phage library make this assay much more scalable (essentially a mass-serological assay) than other high throughput antibody detection platforms, such as protein microarrays or semi-high throughput Luminex immunoassays, which necessitate cloning and expression of recombinant proteins. Nonetheless, PhIP-Seq largely captures antibody binding only to linear epitopes and may fail to detect important binding specificities to conformational antigens present in more traditional assays. On the flip side, the peptide-level sequencing data produced from the PhIP-Seq platform offer a unique opportunity to deconvolute complex patterns of antibody cross-reactivity.

Previous VirScan studies have demonstrated a correlation between increasing age and viral exposure for humans from multiple countries—likely due to generation of persistent, long-lived antibodies from repeated exposures to common non-lethal viruses (e.g., herpesvirus and respiratory syncytial virus) over time (26). As a technology, PhIP-Seq has also been previously used to elucidate the destructive effects of measles infections on broad immunological memory (35) and the cross-reactivity of COVID-19 patient sera to other coronavirues [CoVs (36)], including those derived from animals (37). To date, use of VirScan has been largely concentrated in human and model animal systems (38, 39), but see (40); however, the technology holds enormous promise for application to wildlife zoonotic surveillance systems in the future.

Here, as our primary objective, we use the VirScan platform to profile antiviral antibodies to over 200 human viruses in serum samples derived from two Chiropteran hosts, *Pteropus alecto*, the black flying fox, and *Eonycteris spelaea*, the cave nectar bat. *P. alecto* is a large, nectarivorous and frugivorous bat native to Australia, Papua New Guinea, and Indonesia. *Pteropus alecto* has been previously identified as a reservoir for zoonotic Australian bat lyssavirus (41, 42) and Hendra henipavirus (4, 43, 44). *Pteropus alecto* also co-roost with other bat species, live in close proximity to humans and domesticated wildlife (45, 46), and, in some countries, are hunted for human consumption, offering ample opportunities for cross-species transmission (47). *Eonycteris spelaea* is widespread across Southeast Asia and also known to host a high abundance of viral pathogens (44). Nonetheless, due to the small sample size of the *E. spelaea* dataset, we focus the majority of our analyses on *P. alecto*. As a secondary objective, we use VirScan to investigate how viral exposures covary with age and body condition in these bats. Finally, we outline recommendations for application of PhIP-Seq technology to viral surveillance in wild mammals more broadly, and bats in particular.

## Methods

### Overview and sampling

We obtained serum samples from 77 *P. alecto* and 5 *E. spelaea* bats. *Pteropus alecto* bats were previously wild, free-ranging bats that were presented to wildlife rehabilitation clinics following injury, and subsequently admitted for treatment (40). Other than these injuries, all bats were considered, anecdotally, to be of generally good health at initial intake, though no demographic or morphometric data were recorded. The majority (*n* = 71) of *P. alecto* bats were recovered in South-East Queensland, Australia, whereas the remaining bats (*n* = 6) were from North Queensland and transported to Brisbane. *Pteropus alecto* bats were admitted to rehabilitation clinics between January 2014 and October 2017.

Following intake, *P. alecto* bats (*n* = 77) were temporarily housed in a carer’s facility for anywhere between 1 day to 6 weeks, after which those that were determined to have irreparable physical damage that precluded their release to the wild were euthanized for this study. Four bats (Appendix S1; Supplementary Table S1) were treated with anti-fungal therapy for 2 weeks throughout rehabilitation, though treatment was withdrawn at least 7 days prior to euthanasia and concomitant sampling. Demographic information and morphometric data were then recorded for all bats at euthanasia, including: sex, age class (as juvenile, subadult, and adult based on size and reproductive characteristics) (48), forearm length (mm), body mass (g), and body condition (poor, fair, good, excellent; based on palpation of the pectoral muscle mass; Supplementary Table S1).

Handling, euthanasia, and processing of *P. alecto* was approved by Queensland Animal Science Precinct & University of Queensland Animal Ethics Committee (AEC# SVS/073/16/USGMS) and the Australian Animal Health Laboratory (AAHL) Animal Ethics Committee (AEC# 1389 and AEC# 1557). Blood was collected from all bats by puncturing the cephalic vein of the wing. After euthanasia, bats were necropsied, and any adverse health conditions were recorded. Approximately one-third (29) of *P. alecto* bats demonstrated poor health symptoms ranging from osteomyelitis to slimy wing, a fungal infection of the skin (Appendix 1; Supplementary Table S1) (49). For a subset of our *P. alecto* bats (*n* = 39), a canine tooth was extracted upon euthanasia and sent to Matson’s Laboratory (Missoula, MT^1^) for histological analysis of cementum layers for aging. Bats were assigned an integer age based on *cementum annuli* counts, following previously published methods (28, 50–52).

*Eonycteris spelaea* bats were wild-caught in October 2015 (2) and April 2016 (3), then introduced into custom-made stainless-steel cages (100 cm long × 100 cm wide × 183 cm high) at Duke-National University of Singapore (53). Immediately following capture, bats were anesthetized with isoflurane gas and then euthanized via overdose of sodium pentobarbital at 90 to 100 mg/kg body weight. Blood was then collected by puncturing the cephalic vein of the wing. Animal ethics approval was granted by SingHealth Institutional Animal Care and Use Committee (IACUC; Permit # 2015/SHS/1088 and # 2020/SHS/1582). All bats were handled under the assumption that there might be some biosafety concerns for both the handler and the bats. Therefore, to maximize safe handling of wild bats, researchers wore personal protective equipment (Tyvek suits, gloves, masks, etc.) at all times. Any biological samples were processed according to the biosafety laboratory certification at the University of Queensland and the Duke National University of Singapore as described in the approved AEC and IACUC permits.

### VirScan

*Pteropus alecto* serum samples were serologically profiled using the original VirScan library on a PhIP-Seq platform at the Duke-NUS Medical School in Singapore. *Eonycteris spelaea* samples were serologically profiled using the same library on a PhIP-Seq platform in the Larman lab at Johns Hopkins University in Baltimore, Maryland. Both data sets include a comprehensive report of past or ongoing exposures to each of the >200 viruses included in the original human virome library. For a detailed description of the VirScan library, see other sources (26, 31–33, 40). Briefly, protein sequences from whole genomes of 206 virus species and 1,000 virus strains were divided into overlapping 56mer peptide fragments and synthesized commercially as oligo pools. An oligonucleotide library encoding the peptides was synthesized and cloned into T7 bacteriophage. Serum from bat samples were then incubated with the library and immunoprecipitated using protein A/G coated magnetic beads. Unbound phages were washed away, and only bound phages remained. The precipitate was then amplified by PCR and parallel sequencing of the phage library used to quantify enrichment due to antibody binding. Ultimately, PhIP-Seq produces peptide-level antibody reactivity to all viruses for each serum sample (35, 54).

### Quantification of viral hits

Following previously published methods, we calculated Z-scores corresponding to peptide enrichment as compared to a suite of negative control reactions (beads-only, no serum) (35, 54). Briefly, peptides were rank ordered according to peptide enrichment in beads-only reactions based on NGS read counts. Then epitopes, with identical or very similar baseline abundance were grouped into bins of 300 epitopes, as previously described (35). For each serum sample, the top and bottom 5% of epitopes, binned by NGS read counts, were removed from the analysis and the mean and standard deviation of the middle 90% were used to determine the null distribution. Z-scores for each peptide were calculated by comparing the NGS read counts for a given peptide in a single sample with the sample-specific null distribution (35).

Because many different viruses share overlapping peptide fragments, we next sought to identify past virus exposures in a given sample by quantifying the hierarchy of peptide enrichment among closely-related antigens. To this end, we used the AntiViral Antibody Response Deconvolution Algorithm (AVARDA) developed by Monaco et al. (55). AVARDA rank orders potential virus exposures based on the number of enriched peptides per virus in a single sample, then uses a database of amino acid overlaps among peptides to priority rank enriched peptides shared between related viruses and determine the most likely viral exposure to have elicited the corresponding antibody response (55). The AVARDA pipeline requires several parameter inputs prior to initialization: a value of p-threshold delineating the significance threshold for positive enrichment of a z-score based on its deviation from the null distribution, a bh-threshold that corrects this value of p for the assumption of multiple simultaneous hypothesis testing as many viruses are queried at once, a z-threshold that identifies the lower limit z-score that the model considers to be reactive, and an x-threshold corresponding to the minimum number of peptide enrichments per virus required for designation as a positive “hit.” We set the thresholds within AVARDA at standard values used in previously published work: value of *p*<0.01, Z_threshold = 10, x_threshold = 3, bh_threshold = 0.05 (26, 35, 55). For further descriptions on cut-off values, see (55) and drmonaco/AVARDA.git.

All raw data, alignment and peptide count scripts (bowtie2 and samtools), z-score binning pipeline, and code for downstream analyses and plotting are available in our open-access, GitHub repository.^2^

### Statistical analysis

**Figure 1:**
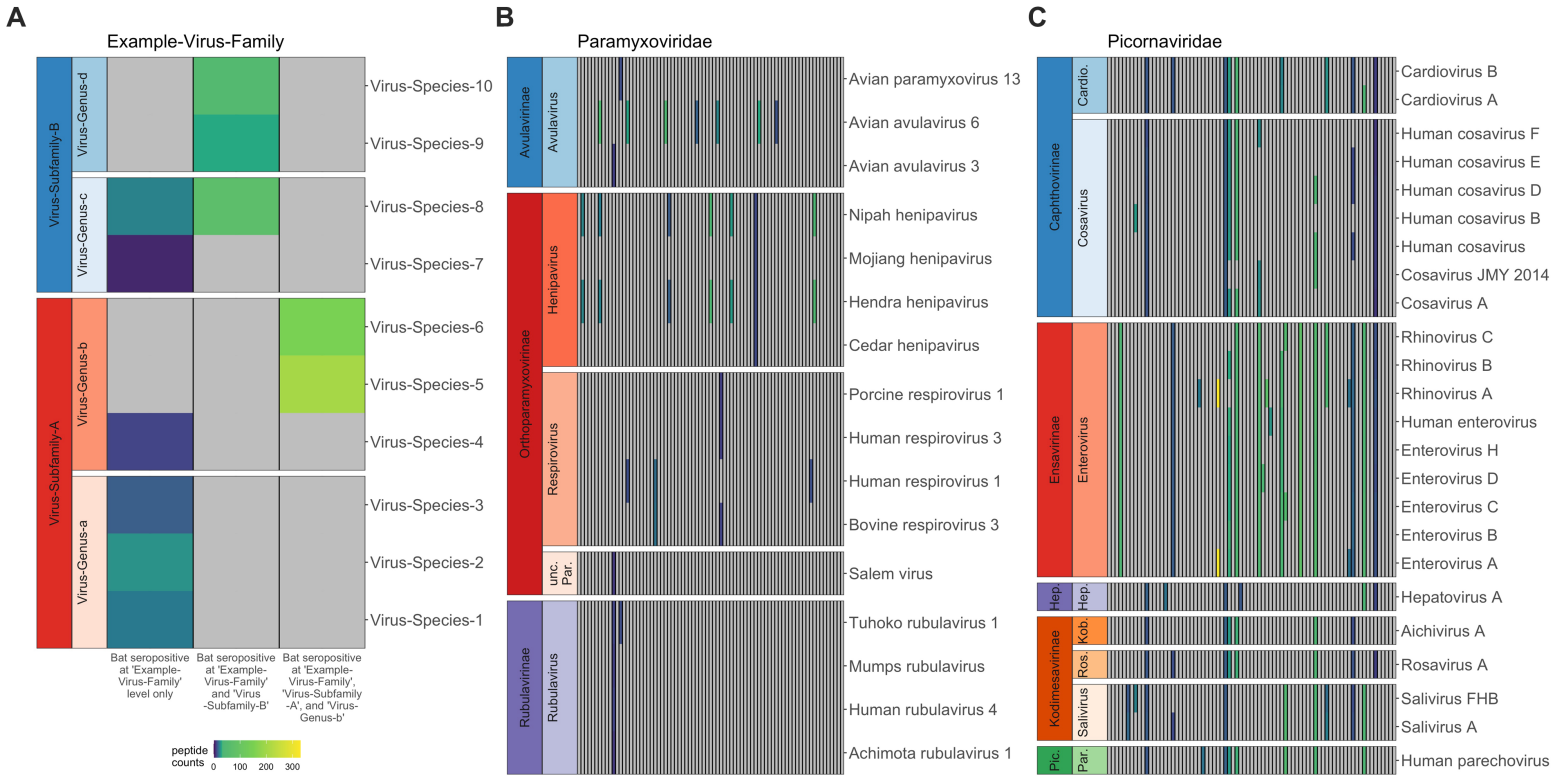
Co-infection or broad binding of antibodies to closely-related antigens across the peptide library for *Pteropus alecto*. When antibodies in serum from a single individual bound antigens from multiple subfamilies in the same virus family, we interpreted this as a single exposure to one family; likewise, when antibodies bound antigens from multiple genera within the same subfamily, we interpreted this as a single exposure to that entire subfamily; and when antibodies bound antigens from multiple virus species in the same genus, we interpreted this as a single exposure to that genus **(A)**. Viral species (y-axis) are sorted according to viral subfamily and genus, thus the related viruses (e.g., within the same clade) are clustered together. Each column represents data for a distinct individual, and gray panels indicate 0 peptide hits; colors represent positive hit values, following legend. The human-focused PhIP-Seq library effectively distinguishes infections in bats, though it does better within bat-specific clades **(B)** than within human-specific clades **(C)**, where we see a lot of antibody cross reactivity across genera.

Statistical data analysis was performed in RStudio v1.3.959 (R Core Team, 2020). As our primary objective, we broadly explored the past viral exposures of *P. alecto* and *E. spelaea.* Because bat sera were assayed against a human-focused peptide library [VirScan (26)], we chose to conservatively tabulate exposures at broader virus family, subfamily, and genus levels, assuming that enriched peptides corresponding to multiple viral species within the same clade were more likely to reflect broad binding of antibodies generated from a single virus exposure to closely-related antigens, rather than true instances of multiple exposure to closely-related but distinct virus genotypes. Thus, when antibodies in serum from a single individual demonstrated binding to multiple virus species (*n* ≥ 2) within the same genus, we interpreted this as a single exposure to one virus within the genus, resulting in antibodies capable of binding multiple closely-related epitopes (26). Less commonly, when antibodies in serum from one individual bound epitopes across different viral genera within the same virus subfamily, we scored this exposure at only the subfamily level, and when antibodies in serum from one individual bound epitopes across different viral subfamilies within the same virus family, we scored this exposure at only the family level (Figure 1A). We acknowledge the potential loss of detail on co-exposure histories for closely-related viruses; however, we considered this more conservative approach appropriate as an initial step until additional bat-virus specific peptides can be incorporated into future assays. Using these criteria, we calculated the seroprevalence of each viral family, subfamily, and genus for both bat species and compared the number of exposures to diverse viral genera with the number of exposures to specific viral species previously reported for humans (26).

Secondarily, we explored how the quantity of viral peptide hits, total virus exposures, and serostatus to particular viral genera covaried with key morphological variables in *Pteropus alecto*. First, we calculated the regression of body mass (in grams, as recorded upon euthanasia) onto forearm length (in mm) for adult and subadult bats, then extracted the residuals (*n* = 59 *P. alecto*, *n =* 5 *E. spelaeae*; Supplementary Figure S1). For full-grown bats, the mass: forearm residual can give a rough estimate of body condition or nutritional health status (56), as individuals with higher masses than predicted by structural size (e.g., forearm) have positive residuals (corresponding to good body condition), while individuals with lower masses than predicted by structural size have negative residual values (corresponding to poor body condition). To assess interactions between bat body condition and past viral exposures (both recorded at the time of euthanasia), we fit a generalized linear model in the binomial family to the response variable of serostatus (0 or 1) as explained by the predictor variable of the interaction of mass: forearm residual with viral genera, including a random effect of bat ID and a Bonferroni correction to minimize type I error in the simultaneous evaluation of multiple (*n* = 57) hypotheses. Subsequently, we applied two generalized linear models in the poison family to a subset of the data (*n* = 39, *P. alecto*) for which we possessed bat ages from *cementum annuli* quantification. In these models, we explored the combined effect of mass: forearm residual and age on (a) the total number of viral peptide hits computed by AVARDA and (b) the total number of viral exposures, computed using the output from the AVARDA pipeline followed by our more rigorous filtering criteria for each bat. Finally, to elucidate viral dynamics, we plotted age-seroprevalence trends for all viral genera identified in *P. alecto* serum. Due to the low sample size of bats aged via *cementum annuli* analysis (*n* = 39, *P. alecto*), we binned age classes together such that bats aged as 2 and 3 years old were binned into 2.5 years, bats 4 and 5 years as 4.5 years, and so on, in order to view patterns in the data (Appendix 1). The limited extent of the aged dataset precluded fitting of any mechanistic transmission models to age-seroprevalence data—though we consider the transmission implications of the observed trends in our discussion.

## Results

Following application of AVARDA to PhIP-Seq Z-score data, we first used heatmaps to visualize raw peptide hit counts across all viral families, subfamilies, and genera for individual bats [Supplementary Figure S2, *P. alecto* (*n* = 77) and Supplementary Figure S3, *E. spelaea* (*n* = 5)]. As expected, we observed more frequent occurrences of multiple epitope binding within the same viral genus or subfamily, likely indicating antibody binding across closely-related viral peptides in our library (Figure 1A). Our PhIP-Seq platform more readily distinguished exposures to peptides derived from virus clades known to circulate in bats (and, in particular, those viruses already known to circulate in *P. alecto*) than to peptides derived from viruses known only to circulate in humans. For example, in general, VirScan effectively differentiated exposure to known bat henipaviruses, Hendra and Nipah, from exposure to less closely-related Mojiang and Cedar henipaviruses (Figure 1B) but was unable to differentiate exposure among closely-related human enteroviruses, or even sometimes more distantly-related human picornaviruses, such as cosaviruses and cardioviruses (Figure 1C). In reality, an individual bat’s seropositivity to a wide range of human picornaviruses likely reflects cross-reactivity in antibodies raised against one or several bat picornaviruses not currently included in the VirScan library. Additionally, VirScan distinctly differentiated antibodies to both Marburg and Ebola filoviruses separately in serum from different *P. alecto* individuals (Supplementary Figure S1), supporting prior reports of co-circulation of disparate Ebola- and Marburg-like filoviruses that generate distinct antibodies in wild-caught *P. alecto* from Australia (57). In the case of bat-associated lyssaviruses, VirScan was largely ineffective at differentiating exposures among diverse virus species (Supplementary Figure S1). Prior studies suggest that lyssaviruses may be particularly dependent on conformational epitopes for cell entry and receptor interaction, suggesting that Virscan may be less effective as a method for differentiating lyssavirus antibodies than for other viruses (58). Nonetheless, in two cases, VirScan effectively identified that antibodies in *P. alecto* serum indicated prior exposure to a lyssavirus within the more closely related phylogroups I, II, and III—but not within the highly divergent phylogroup IV [Lleida/Ikoma viruses (59–61)]. In two other cases, the assay effectively signified a lack of exposure to New World bat RABV lyssaviruses but nonetheless retained cross reactivity across all Old World bat lyssaviruses across all known phylogroups (62).

**Figure 2:**
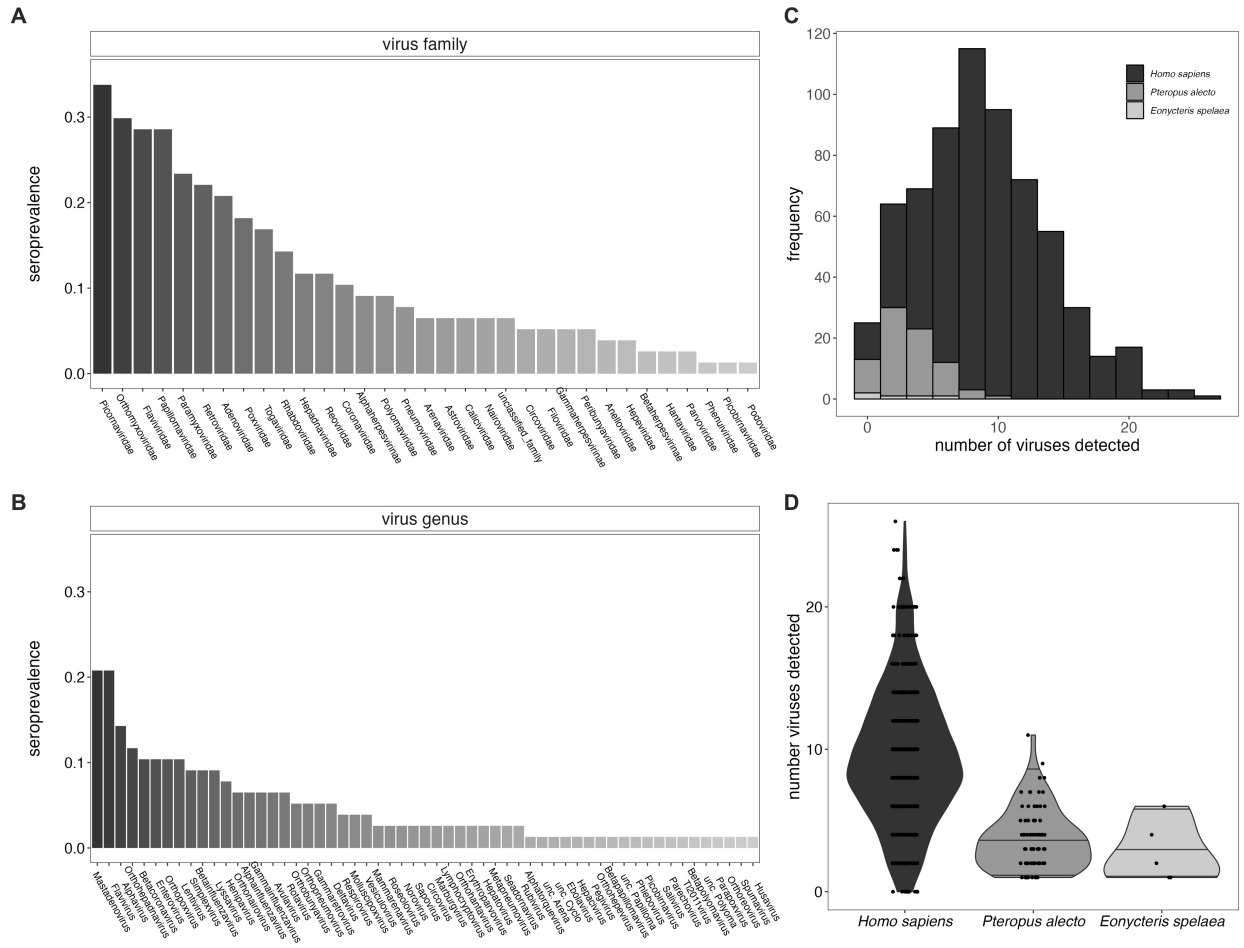
Seroprevalence for viral families **(A)** and viral genera **(B)** for *P. alecto* bats. We identified viral hits to 57 genera which fall within 33 different viral families. **(C)** Histogram of the distribution of the total number of virus exposures as determined in *P. alecto* samples vs. *E. spelaea* samples, as compared with previously reported estimates for *Homo sapiens* (26); **(D)** data recomputed to demonstrate interquartile range. The total number of exposures distinguishable at the level of genus for bats were fewer than previously described to species for humans.

In *P. alecto* samples (*n* = 77), we identified preliminary evidence of prior viral exposure to 57 specific viral genera, 41 subfamilies, and 33 different families (Figures 2A,B). In *E. spelaea*, we identified evidence of prior viral exposure to nine viral genera, 14 subfamilies, and 12 different viral families. Many of the genera identified in *P. alecto* and *E. spelaea* (*n* = 32 and *n* = 9, respectively; Figure 2B) represent the first record of antibodies to these viruses in these bat species—though future work should seek to validate these findings with alternative confirmatory serological methods. Nonetheless, VirScan identified putative evidence of prior exposure to multiple viral genera in *P. alecto* serum that represent the first record of association between these viruses and this bat host: alphatorquevirus, alphavirus, betapapillomavirus, betapolyomavirus, circovirus, deltavirus, erythroparvovirus, hepacivirus, hepatovirus, husavirus, lymphocryptovirus, mammarenavirus, metapneumovirus, mulluscipoxvirus, norovirus, orthohepadnavirus, orthohepevirus, orthopoxvirus, orthoneumovirus, parapoxvirus, parechovirus, pegivirus, phlebovirus, picobirnavirus, roseolovirus, rotavirus, rubivirus, salivirus, sapovirus, seadornavirus, spumavirus, and vesiculovirus.

Furthermore, we identified likely exposure to a mean of 3.69 viral genera per individual for *P. alecto* (Figures 2C,D, median = 3) and 2.8 (median 2) viral genera per individual for *E. spelaea*. While, respectively, these values correspond to approximately one-third and one-fifth the number of viral exposures previously identified in an average adult human [mean = 9.51, median = 10, (26)], this discrepancy likely reflects the reduced specificity of our assay for bat-specific viruses, such that we were able to quantify exposures to only a genus level, or lower levels of circulating antibodies in bats in general. As a result, our detection criteria likely overlooked multiple exposures to closely-related bat virus strains that are absent from the VirScan library; multiple exposures to closely-related virus strains feature heavily in the average number of exposures previously calculated from human datasets (26).

**Figure 3:**
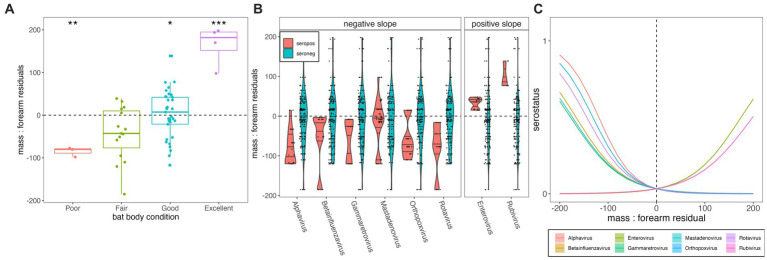
**(A)** Mass:forearm residual (y-axis) by body condition score (x-axis); raw data are plotted as points with the interquartile range and median of each category indicated by the upper, lower, and middle bars of the boxplot. Statistically significant categorical predictors of mass:forearm residual by linear regression are indicated by stars (*p < 0.05, **p < 0.01, and ***p < 0.001). **(B)** Distribution of mass:forearm residual (y-axis) in seropositive (orange) vs. seronegative (blue) individuals, across diverse viral genera (x-axis). Genera are grouped according to statistically significant interactions demonstrating negative (left) and positive (right) associations between seropositivity and mass:forearm residual, as determined via generalized linear mixed effects regression and subsequent Bonferroni correction **(C)**. Interaction plot of the relationship between serostatus (1 = seropositive; 0 = seronegative) and mass:forearm residual across 8 viral genera that demonstrated significant associations. Mass:forearm residuals by serostatus and resulting slopes from generalized linear regression are plotted for all viruses tested, including those for which no significant interaction was demonstrated, in Supplementary Figures S4, S5. Translucent shading (very narrow) corresponds to 95% confidence intervals by standard error.

Total body mass at time of euthanasia (grams) varied positively with forearm length (mm) for *P. alecto* (*n* = 59, adult = adj *R*^2^ = 0.386, *p* < 0.01; subadult = adj *R*^2^ = 0.453, *p* = 0.005; Supplementary Figure S1). Juveniles (*n* = 4, *P. alecto*) were excluded from our analysis, as young bats might still be growing structurally (56). Body mass residuals matched our qualitative scoring metric of “condition” (poor, fair, good, excellent) where those marked as in poor condition had the lowest median mass residuals (*p* < 0.01; Figure 3A) and those marked as in excellent condition had the highest mass residuals (*p* < 0.0001). Generalized linear model results indicated that body condition does vary significantly with serostatus for some viral genera—in most cases (Alphavirus, Gammaretrovirus, Betainfluenzavirus, Mastadenovirus, Orthopoxvirus, and Rotavirus), showing a negative association between seropositivity and body condition, but in a few cases (Enterovirus and Rubivirus), showing a positive association (Supplementary Figures S4, S5; Figures 3B,C). Seropositivity for viral families known to circulate naturally in wild bats (e.g., coronaviruses, henipaviruses, lyssaviruses, filoviruses) showed no significant associations with either lower or higher mass: forearm residuals (Supplementary Figures S4, S5). As PhIP-Seq measures history of exposure, rather than active infection, these findings do not offer evidence of any hypothesis for either a lack or presence of significant pathogenicity for these viruses in their bat hosts.

**Figure 4:**
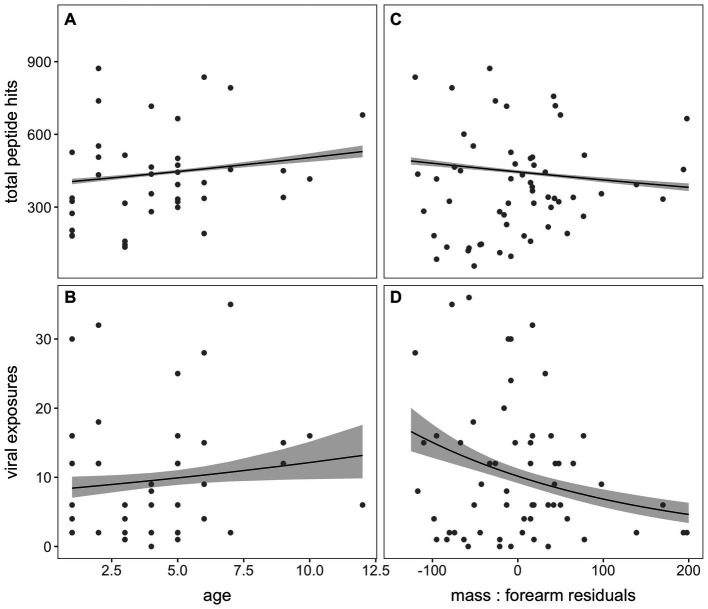
Total peptide hits and the number of viral exposures, computed through AVARDA, demonstrated a positive association with age in years **(A,B)** and a negative association with mass: forearm residuals **(C,D)** in *P. alecto* bats, indicating subtle morbidity effects of frequent virus exposure on bat hosts or an increased likelihood of infection in underweight bats.

For the subset of bats aged via *cementum annuli* quantification in our dataset (*n* = 39; Appendix 1), we observed a significant positive association between age and both total peptide hits across the entire VirScan library (*p* < 0.001) and the total number of viral exposures defined by our criteria following AVARDA (*p* = 0.035; Figures 4A,B, respectively). Additionally, we identified a significant negative association between mass: forearm residual scores (where higher scores are a measure of “healthier” bat body condition) and both total peptide hits and total viral exposures (*p* < 0.001, for both; Figures 4C,D, respectively).

**Figure 5:**
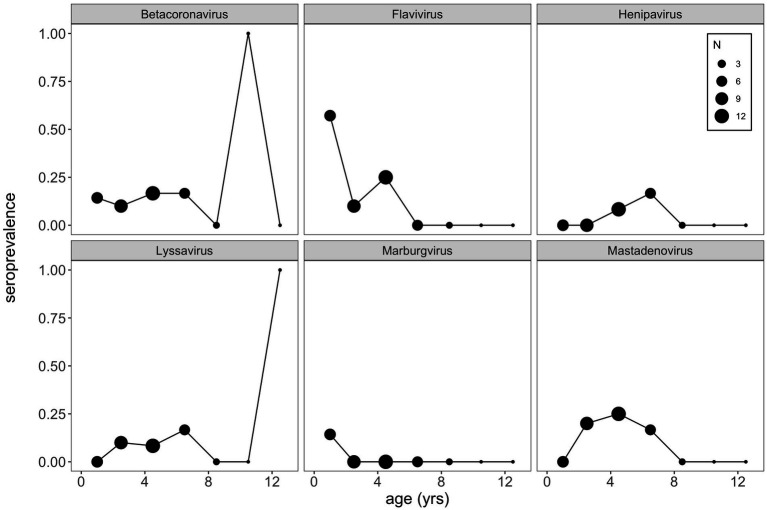
VirScan can be used as a tool to explore age-seroprevalence patterns, which are useful in inferring pathogen dynamics. Here, we see little evidence of increasing seroprevalence with age (a classic signature of SIR dynamics), which might suggest that waning antibodies and cycles of immunity may underpin bat virus dynamics more broadly. Importantly, a bat-specific VirScan library might elucidate these trends more clearly. Note that we binned age classes together such that bats aged as 2 and 3 years old were binned into 2.5 years, bats 4 and 5 years as 4.5 years, and so on, in order to view patterns in the data.

Finally, using this same aged subset of our *P. alecto* data, we plotted age-seroprevalence trends across all viral genera for which seropositives were identified in our selection criteria (Figure 5; Supplementary Figure S6). Age-seroprevalence patterns varied across diverse genera and were challenged by small sample sizes. High seroprevalence in juveniles was observed for a few viral genera (e.g., flavivirus, enterovirus, and orthopoxvirus), followed by rapidly waning immunity, suggesting the potential for inherited maternal antibodies in some cases. Seropositivity to select viral genera (e.g., betacoronavirus, lyssavirus, orthopneumovirus, orthopoxvirus, and pegivirus) was observed in a few older age (10+ years) individuals, despite broad trends of declining seroprevalence in later life.

## Discussion

Here, we explore the history of broad viral exposure in *Pteropus alecto* and *Eonycteris spelaea* using a novel application of PhIP-Seq technology. Our work shows that bats are exposed to a wide variety of viruses, including many of known zoonotic potential (3). Importantly, our analysis demonstrates that PhIP-Seq has great potential to be useful for viral surveillance in bats, even when limited to a library not designed with bat-specific viruses in mind. Nonetheless, we observed that seropositivity to specific viral genera was most easily identifiable for viral taxa known to circulate naturally in bat hosts (e.g., paramyxoviruses, henipaviruses, filoviruses, and coronaviruses). For viral taxa largely represented by human viruses only in our VirScan library (e.g., adenoviruses and picornaviruses), our inference was limited to recognition of seropositivity at the level of virus subfamily or family only. Particularly, this VirScan assay successfully differentiated between past exposures to the closely related bat virus genera, ebolavirus and marburgvirus (Figure 1B), and even distinguished Hendra/Nipah virus exposure from other peptides within the bat-infecting henipavirus genus (Figure 1B). However, we were sometimes unable to serologically distinguish exposures among subfamilies, likely in large part because certain viruses (e.g., picornaviruses) included in the VirScan library are entirely human in origin. Additionally, as previously noted as a limitation of VirScan (31), the use of linear epitopes in the VirScan library is likely to influence detection ability, especially for certain taxa where antibody binding is particularly conformational in the binding epitope (e.g., lyssaviruses).

Previous studies have noted that bats typically harbor numerous viruses, but without showing any clinical signs of disease (10, 21). Indeed, with the exception of rabies, and related lyssaviruses, and potentially, Lloviu filovirus and Tacaribe arenavirus (11–15), viral infection has not typically been observed to cause mass loss in bats (63, 64). Nonetheless, most studies reporting infection surveillance in the wild or experimentation in the laboratory have only examined mass change on short timescales following challenge with a single pathogen. Here, as a secondary objective, we explored how viral exposures covary with age and body condition. We observed an overall negative relationship between mass: forearm residual—commonly used as a marker of bat health or malnutrition—and both the total number of viral peptide hits experienced across a bat’s lifetime and the total number of prior exposures. It is important to note that the *P. alecto* bats considered in our study spent anywhere from 1 day to <6 weeks in captivity prior to collection of blood for VirScan and recording of mass for analysis. While viral exposures would be unlikely to change substantially during solitary rehabilitation, bat mass may have been more easily modulated under captive conditions, particularly for individuals held for longer periods of time. Unfortunately, poor records from the rehabilitation center precluded quantitative assessment of the impact of these conditions on our analyses. Nonetheless, we hypothesized that bats would be more likely to have gained than to have lost mass in captivity, thus most likely dampening any association with prior viral exposure. Despite these limitations, we still observe a significant negative relationship between mass: forearm residual and virus exposure, suggesting either some subtle negative effects of continued or repeated viral infection on bat health over time, or a heightened susceptibility to viral exposure in individuals with poor nutritional status. While patterns arising from this PhIP-Seq analysis are intriguing, seropositivity to key viral taxa should nonetheless be confirmed with more traditional ELISA- or Luminex-based serological approaches and validated across larger sample sizes and with other bat species. Results should also be compared with molecular detection approaches, like metagenomic next generation sequencing (mNGS) and polymerase-chain reactions (PCR), to assess the effects of current infection on bat health, rather than past exposures alone.

Coupling serology and host age data into age-seroprevalence curves allows for retrospective estimation of the time since infection, a practice which has been previously employed to help disentangle the transmission dynamics of numerous wildlife and human pathogens (27, 28, 65–70). In classic cases in which viral infection confers perfect, lifelong immunity, we would expect to see patterns of monotonically increasing seroprevalence with age, as individuals in older age cohorts accumulate a steadily increasing cumulative hazard of exposure with advancing age. In cases in which antibody levels wane with time, allowing individuals to become seronegative and return to susceptible status, we would expect to see an age-seroprevalence pattern of increasing seroprevalence in early ages, followed by a plateau in later age classes (because immune individuals constantly wane back to susceptibility but concurrently re-encounter a hazard of re-infection). Prior work in bat virus systems has fit dynamical transmission models to age-seroprevalence curves for henipaviruses and lyssaviruses in African *Eidolon helvum* bats (27) and to henipaviruses in Madagascar *Eidolon dupreanum* bats (28). Both studies identified patterns of waning maternal immunity in neonates and increasing seroprevalence in early age years—which plateaued in the African system (27) and declined in older age individuals in the Madagascar system (28). Respectively, data were best captured by transmission assumptions of waning immunity and reinfection (27) or waning antibodies but persistent cell-mediated or innate immunity (28). The sparsity of aged *P. alecto* (*n* = 39) in our dataset precluded fitting of transmission models to age-seroprevalence curves recovered for any viruses surveyed on the PhIP-Seq platform. Nonetheless, qualitatively, we observed a near-complete absence of a pattern of increasing seroprevalence with age for almost all viral epitopes in our dataset (Supplementary Figure S6), indicating, as has been previously suggested (71), that transmission dynamics for many bat-borne viruses are likely complex and may not follow the classic Susceptible-Infectious-Recovered paradigm. Application of VirScan to serum collected in part with longitudinal surveillance efforts offers an opportunity to elucidate these transmission dynamics for multiple bat viruses simultaneously in the future.

### Future directions

**Figure 6:**
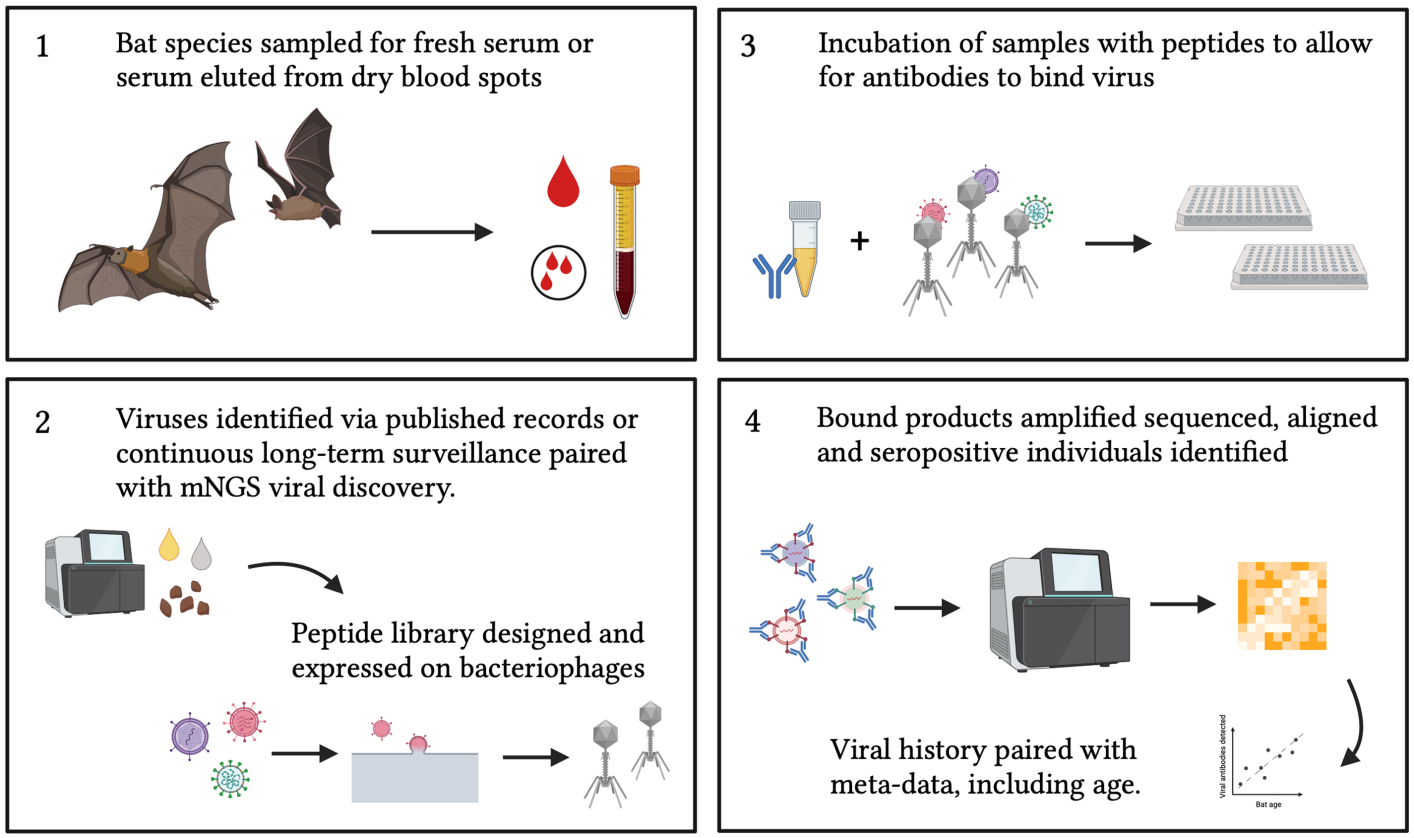
Proposed sampling and design of a bat-specific PhIP-Seq library. Future applications to bat (or wild mammal) samples should also be paired with meta-data that includes age (either tooth analysis, tagging, long-term recapture or epigenetics) and condition to allow insight into viral transmission dynamics. Created with BioRender.com.

Bats have received increasing recognition in recent years due to their role as hosts of multiple viral pathogens (72). Therefore, broad serological surveillance aimed at elucidating the viruses that bats host are of great public and scientific interest (1, 4, 8). Most existing serological surveillance methods, using ELISA or Luminex technology, necessitate targeted assay for specific, pre-defined pathogens; however, as exemplified by the COVID-19 pandemic, zoonotic pathogens can emerge that are previously unknown to science. Metagenomic sequencing offers one powerful tool for unbiased surveillance, but molecular surveillance approaches can only detect active infections, easily missing virus shedding events, which, for bats, may occur only briefly between protracted periods of latent infection (31). VirScan offers a promising alternative for future wildlife surveillance efforts, combining the broad historical outlook of serology—by which to identify both current and past infections—with the broad, multi-pathogen approach of mNGS (34). Nonetheless, though broader in expanse than any previously described serological tool, even VirScan is limited by the current peptide library design (31). To date, most PhIP-Seq platforms have been limited to surveillance of the human virome; future applications of this platform in bat systems should aim to develop a bat-focused peptide library (Figure 6), which can effectively distinguish between multiple exposures to closely-related bat viruses or bat virus strains in a single genus (e.g., sarbecovirus diversity in *Rhinolophus* spp. bat hosts) (73). Because of the simplicity of the PhIP-Seq design (26), a bat-specific or a pan-mammal peptide library could also be easily updated to reflect the near-perpetual additions of newly discovered virus genomes to the literature (73–76). PhIP-Seq thus holds great promise for expanding human biomedical technology for novel surveillance applications in wildlife reservoirs for emerging human diseases.

## Data availability statement

All PhIP-Seq data, metadata, and statistical code used in all analyses are available for download from our public Github repository at https://github.com/brooklabteam/bat-VirScan-public/.

## Ethics statement

The animal study was approved by Handling, euthanasia, and processing of *P. alecto* was approved by Queensland Animal Science Precinct & University of Queensland Animal Ethics Committee (AEC# SVS/073/16/USGMS) and the Australian Animal Health Laboratory (AAHL) Animal Ethics Committee (AEC# 1389 and AEC# 1557). Animal ethics approval was granted by SingHealth Institutional Animal Care and Use Committee (IACUC; Permit # 2015/SHS/1088 and # 2020/SHS/1582). The study was conducted in accordance with the local legislation and institutional requirements.

## Author contributions

EC cleaned and collated the data, ran the analyses, and wrote the manuscript. WC processed VirScan samples, built sequencing library, and assisted with data processing. RF processed all bat samples. AP prepared teeth samples for aging and provided funding for their processing. YL assisted with the alignment and AVARDA pipeline. AI processed the bat samples and co-developed the initial concept with LW. LW oversaw and funded field data collection and VirScan assay of resulting serum samples. CEB assisted in the AVARDA pipeline, refined statistical analyses, created figures, and wrote the manuscript. All authors edited the manuscript. All authors contributed to the article and approved the submitted version.

## Funding

This research was funded by grants to EC (Grainger Bioinformatics Center) and CB (Branco Weiss “Society in Science” fellowship and University of Chicago start-up funds). Work conducted at Duke-NUS was funded by grants from the Singapore National Research Foundation (NRF2012NRF-CRP001-056 and NRF2016NRF-NSFC002-013), the National Medical Research Council (COVID19RF-003 and OFLCG19May-0034). Tooth analyses were supported by a Griffith University New Researcher grant and an ARC DECRA fellowship to AP (DE190100710). AI is funded by a Key grant from the National Science Foundation of Zhejiang Province (Z23C010003). LFW: Work conducted at Duke-NUS was funded by grants from the Singapore National Research Foundation (NRF2012NRF-CRP001-056 and NRF2016NRF-NSFC002-013), the National Medical Research Council (COVID19RF-003 and MOH-OFLCG19May-0003).

## Conflict of interest

HL is an inventor on an issued patent (US20160320406A) filed by Brigham and Women’s Hospital that covers the use of the VirScan technology, is a founder of ImmuneID, Portal Bioscience and Alchemab, and is an advisor to TScan Therapeutics. WC is an employee of the company CoV Biotechnology Pte Ltd.

The remaining authors declare that the research was conducted in the absence of any commercial or financial relationships that could be construed as a potential conflict of interest.

## Publisher’s note

All claims expressed in this article are solely those of the authors and do not necessarily represent those of their affiliated organizations, or those of the publisher, the editors and the reviewers. Any product that may be evaluated in this article, or claim that may be made by its manufacturer, is not guaranteed or endorsed by the publisher.

## Footnotes

^1^ https://matsonslab.com

^2^ https://github.com/brooklabteam/bat-Virscan-public

## Supplementary material

SUPPLEMENTARY FIGURE S1 Relationship between mass (grams; at time of euthanasia) and forearm (mm) of *P*. alecto for adult (top) and subadult (bottom) bats. In both instances, forearm size predicts body mass (*p* < 0.01).

SUPPLEMENTARY FIGURE S2 Co-infection or cross-reactivity for all viral families in the dataset for *Pteropus alecto*. Viral species (y-axis) are sorted according to viral subfamily and genus, thus the related viruses (e.g. within the same clade) are clustered together. Each column represents data for a distinct individual, and gray panels indicate 0 peptide hits; colors represent positive hit values, following legend.

SUPPLEMENTARY FIGURE S3 Co-infection or cross-reactivity for all viral families in the dataset for *Eonycteris spelaea*. Viral species (y-axis) are sorted according to viral subfamily and genus, thus the related viruses (e.g. within the same clade) are clustered together. Each column represents data for a distinct individual, and gray panels indicate 0 peptide hits; colors represent positive hit values, following legend.

SUPPLEMENTARY FIGURE S4 Effects of serostatus on mass:forearm (mass residuals) for all viral genera in the dataset for *P. alecto*. Significant effects of mass residuals on serostatus, as determined by generalized linear mixed effect regression with a Bonferroni correction, varied by genus; eight viral genera demonstrating significant interactions with mass residual are indicated by stars (***).

SUPPLEMENTARY FIGURE S5 Serostatus response to mass:forearm residual, output from generalized linear mixed effects regression. Significant interactions for eight viral genera are indicated by stars (***); all other interactions were not significant.

SUPPLEMENTARY FIGURE S6 Seroprevalence patterns across age in years in *P. alecto*, as determined from *cementum annuli* analyses, for all viral genera hits.
