# Supplementary figures and images for: Applications of VirScan to broad serological profiling of bat reservoirs for emerging zoonoses
Source: Front Public Health. 2023 Sep 22;11:1212018. doi: 10.3389/fpubh.2023.1212018 (PMC10559906; doi:10.3389/fpubh.2023.1212018)

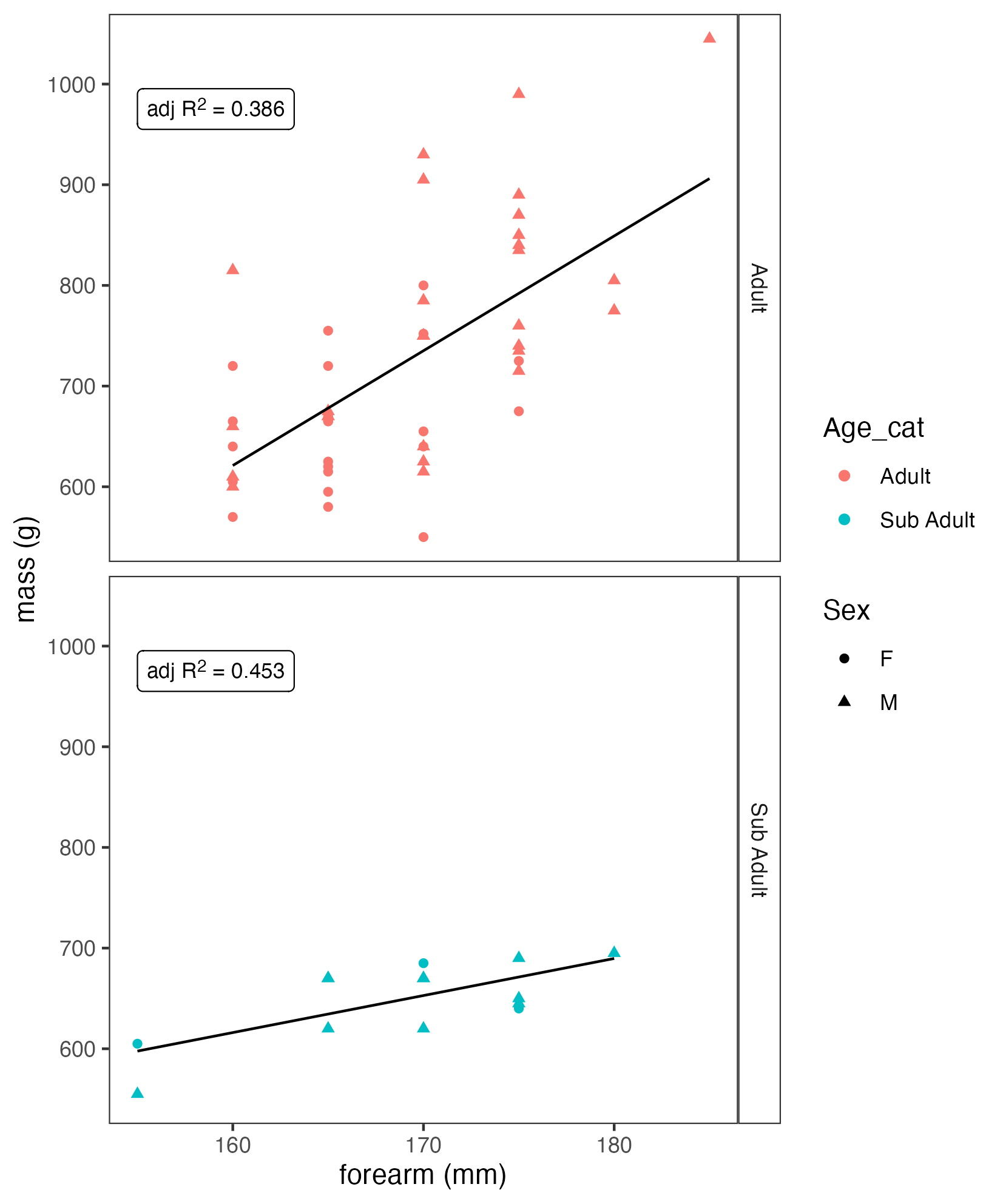

Supplement: SUPPLEMENTARY FIGURE S1 — Relationship between mass (grams; at time of euthanasia) and forearm (mm) of P. alecto for adult (top) and subadult (bottom) bats. In both instances, forearm size predicts body mass (p < 0.01). [file Image_1.JPEG]

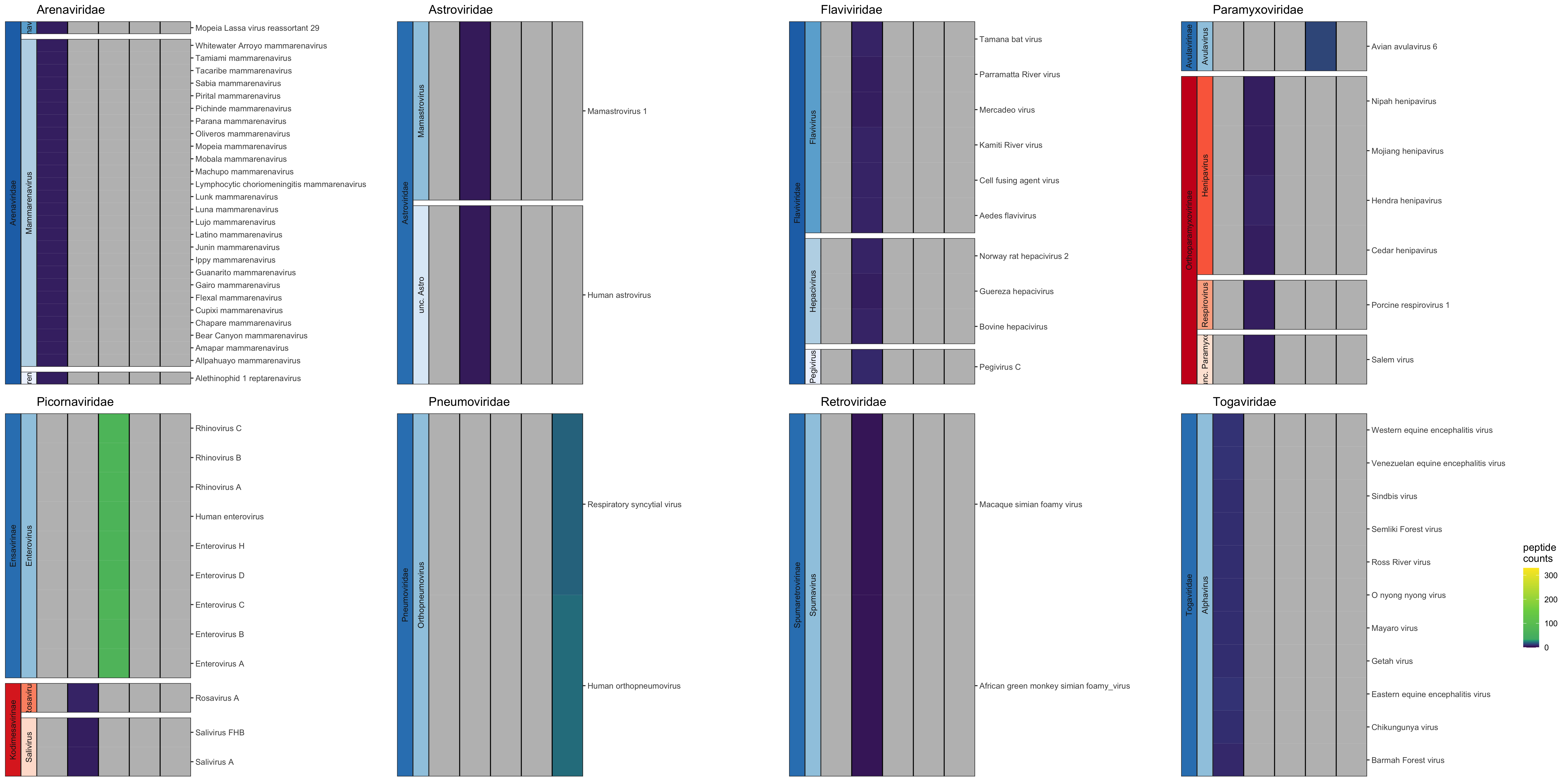

Supplement: SUPPLEMENTARY FIGURE S3 — Co-infection or cross-reactivity for all viral families in the dataset for Eonycteris spelaea. Viral species (y-axis) are sorted according to viral subfamily and genus, thus the related viruses (e.g. within the same clade) are clustered together. Each column represents data for a distinct individual, and gray panels indicate 0 peptide hits; colors represent positive hit values, following legend. [file Image_3.JPEG]

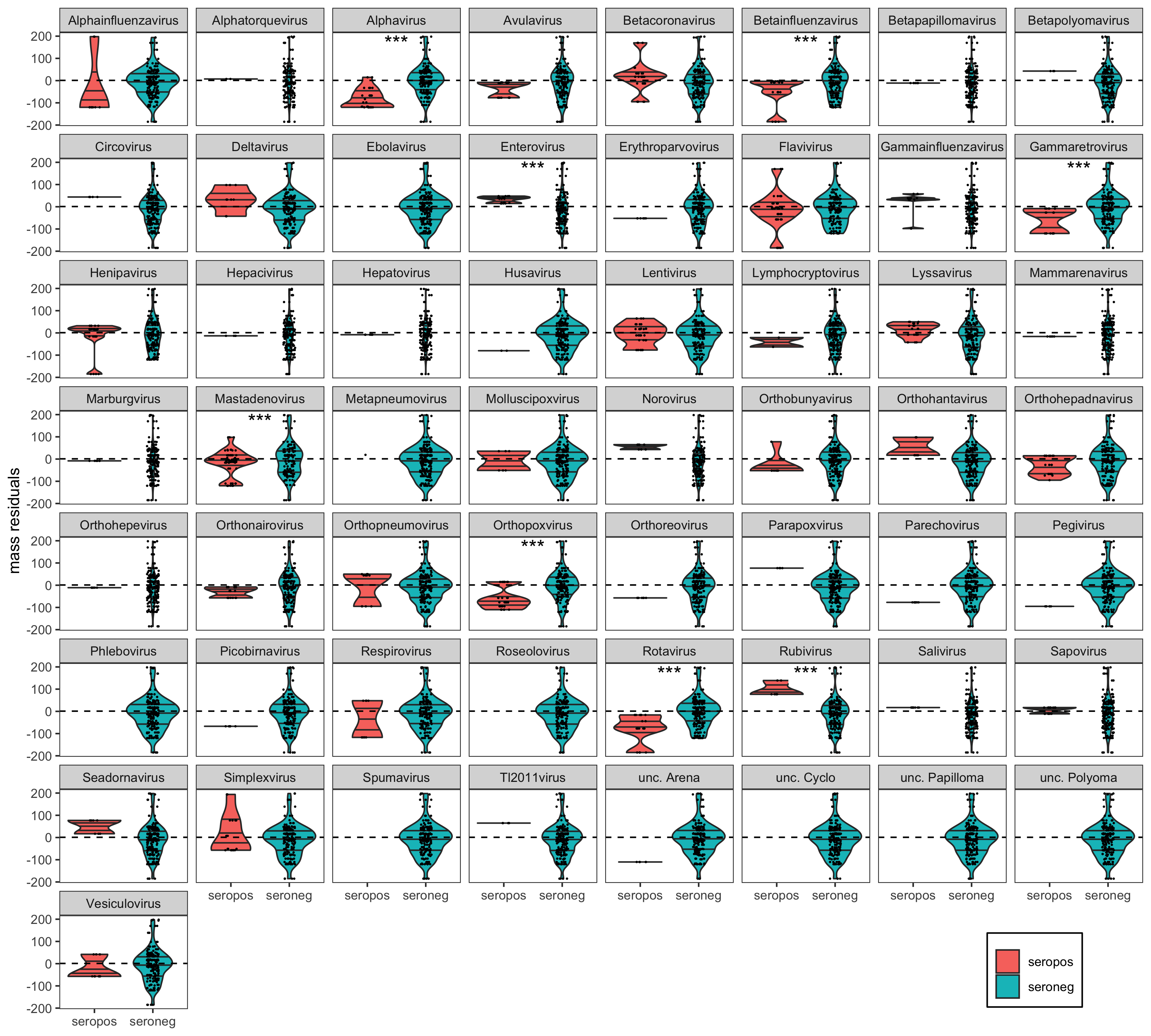

Supplement: SUPPLEMENTARY FIGURE S4 — Effects of serostatus on mass:forearm (mass residuals) for all viral genera in the dataset for P. alecto. Significant effects of mass residuals on serostatus, as determined by generalized linear mixed effect regression with a Bonferroni correction, varied by genus; eight viral genera demonstrating significant interactions with mass residual are indicated by stars (***). [file Image_4.JPEG]

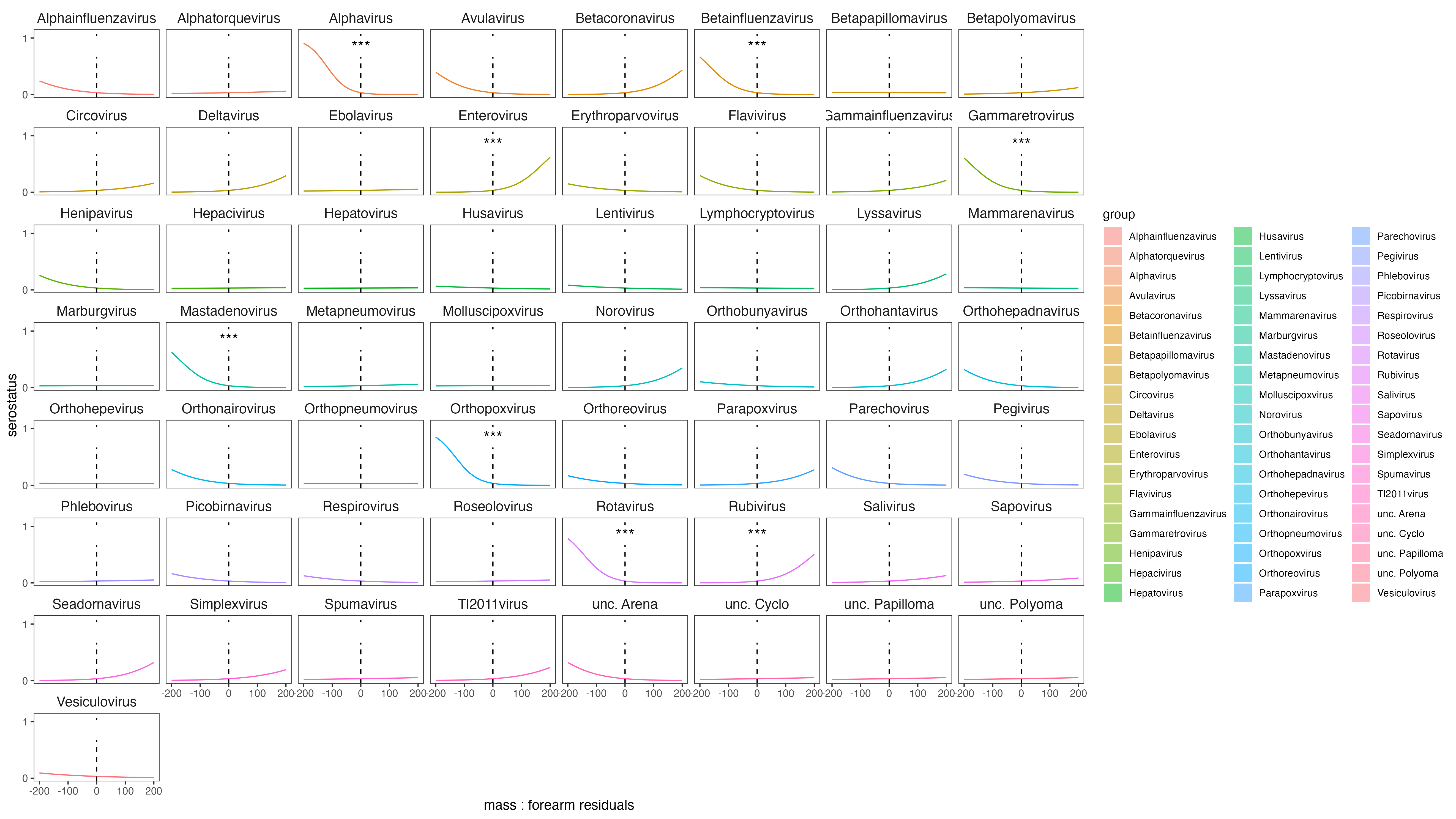

Supplement: SUPPLEMENTARY FIGURE S5 — Serostatus response to mass:forearm residual, output from generalized linear mixed effects regression. Significant interactions for eight viral genera are indicated by stars (***); all other interactions were not significant. [file Image_5.JPEG]

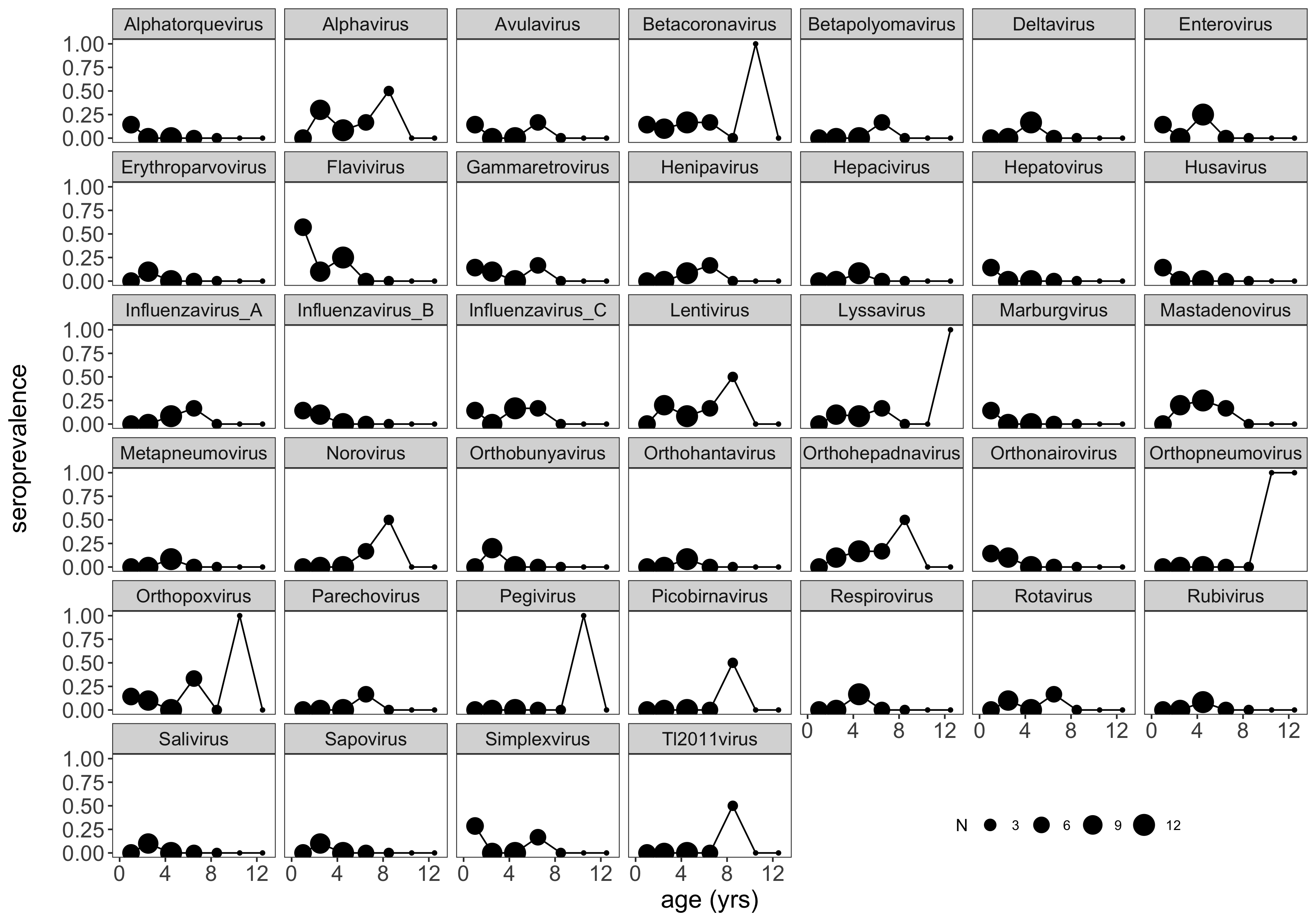

Supplement: SUPPLEMENTARY FIGURE S6 — Seroprevalence patterns across age in years in P. alecto, as determined from cementum annuli analyses, for all viral genera hits. [file Image_6.JPEG]
