# Supplementary material for: Applications of VirScan to broad serological profiling of bat reservoirs for emerging zoonoses
Source: Front Public Health. 2023 Sep 22;11:1212018. doi: 10.3389/fpubh.2023.1212018 (PMC10559906; doi:10.3389/fpubh.2023.1212018)
Supplement: SUPPLEMENTARY FIGURE S2 — Co-infection or cross-reactivity for all viral families in the dataset for Pteropus alecto. Viral species (y-axis) are sorted according to viral subfamily and genus, thus the related viruses (e.g. within the same clade) are clustered together. Each column represents data for a distinct individual, and gray panels indicate 0 peptide hits; colors represent positive hit values, following legend. [file Image_2.PDF]

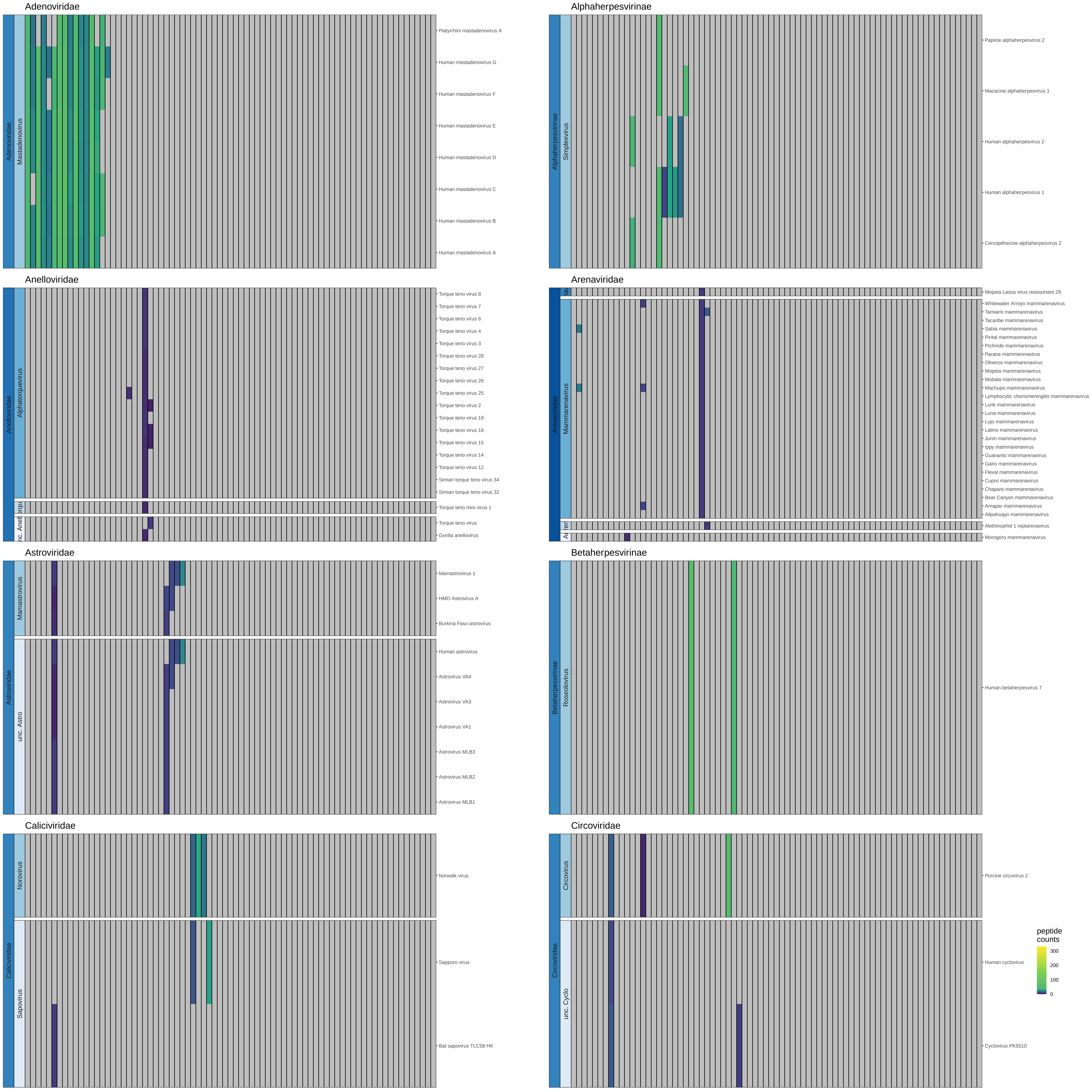

Adenoviridae
Adenoviridae
Mastadenovirus
Platyrrhini mastadenovirus A
Human mastadenovirus G
Human mastadenovirus F
Human mastadenovirus E
Human mastadenovirus D
Human mastadenovirus C
Human mastadenovirus B
Human mastadenovirus A
Alphaherpesvirinae
Alphaherpesvirinae
Simplexvirus
Papiine alphaherpesvirus 2
Macacine alphaherpesvirus 1
Human alphaherpesvirus 2
Human alphaherpesvirus 1
Cercopithecine alphaherpesvirus 2
Anelloviridae
Anelloviridae
Alphatorquevirus
Torque teno virus 8
Torque teno virus 7
Torque teno virus 6
Torque teno virus 4
Torque teno virus 3
Torque teno virus 28
Torque teno virus 27
Torque teno virus 26
Torque teno virus 25
Torque teno virus 2
Torque teno virus 19
Torque teno virus 16
Torque teno virus 15
Torque teno virus 14
Torque teno virus 12
Simian torque teno virus 34
Simian torque teno virus 32
Torque teno mini virus 1
Torque teno virus
Gorilla anellovirus
Arenaviridae
Arenaviridae
Mammarenavirus
Mopeia Lassa virus reassortant 29
Whitewater Arroyo mammarenavirus
Tamiami mammarenavirus
Tacaribe mammarenavirus
Sabia mammarenavirus
Pirital mammarenavirus
Pichinde mammarenavirus
Parana mammarenavirus
Oliveros mammarenavirus
Mopeia mammarenavirus
Mobala mammarenavirus
Machupo mammarenavirus
Lymphocytic choriomeningitis mammarenavirus
Lunk mammarenavirus
Luna mammarenavirus
Lujo mammarenavirus
Latino mammarenavirus
Junin mammarenavirus
Ippy mammarenavirus
Guanarito mammarenavirus
Gairo mammarenavirus
Flexal mammarenavirus
Cupixi mammarenavirus
Chapare mammarenavirus
Bear Canyon mammarenavirus
Amapar mammarenavirus
Allpahuayo mammarenavirus
Alethinophid 1 reptarenavirus
Morogoro mammarenavirus
Astroviridae
Astroviridae
Mamastrovirus
unc. Astro
Mamastrovirus 1
HMO Astrovirus A
Burkina Faso astrovirus
Human astrovirus
Astrovirus VA4
Astrovirus VA3
Astrovirus VA1
Astrovirus MLB3
Astrovirus MLB2
Astrovirus MLB1
Betaherpesvirinae
Betaherpesvirinae
Roseolovirus
Human betaherpesvirus 7
Caliciviridae
Caliciviridae
Norovirus
Sapovirus
Norwalk virus
Sapporo virus
Bat sapovirus TLC58 HK
Circoviridae
Circoviridae
Circovirus
unc. Cyclo
Porcine circovirus 2
Human cyclovirus
Cyclovirus PK5510
peptide counts
300
200
100
0

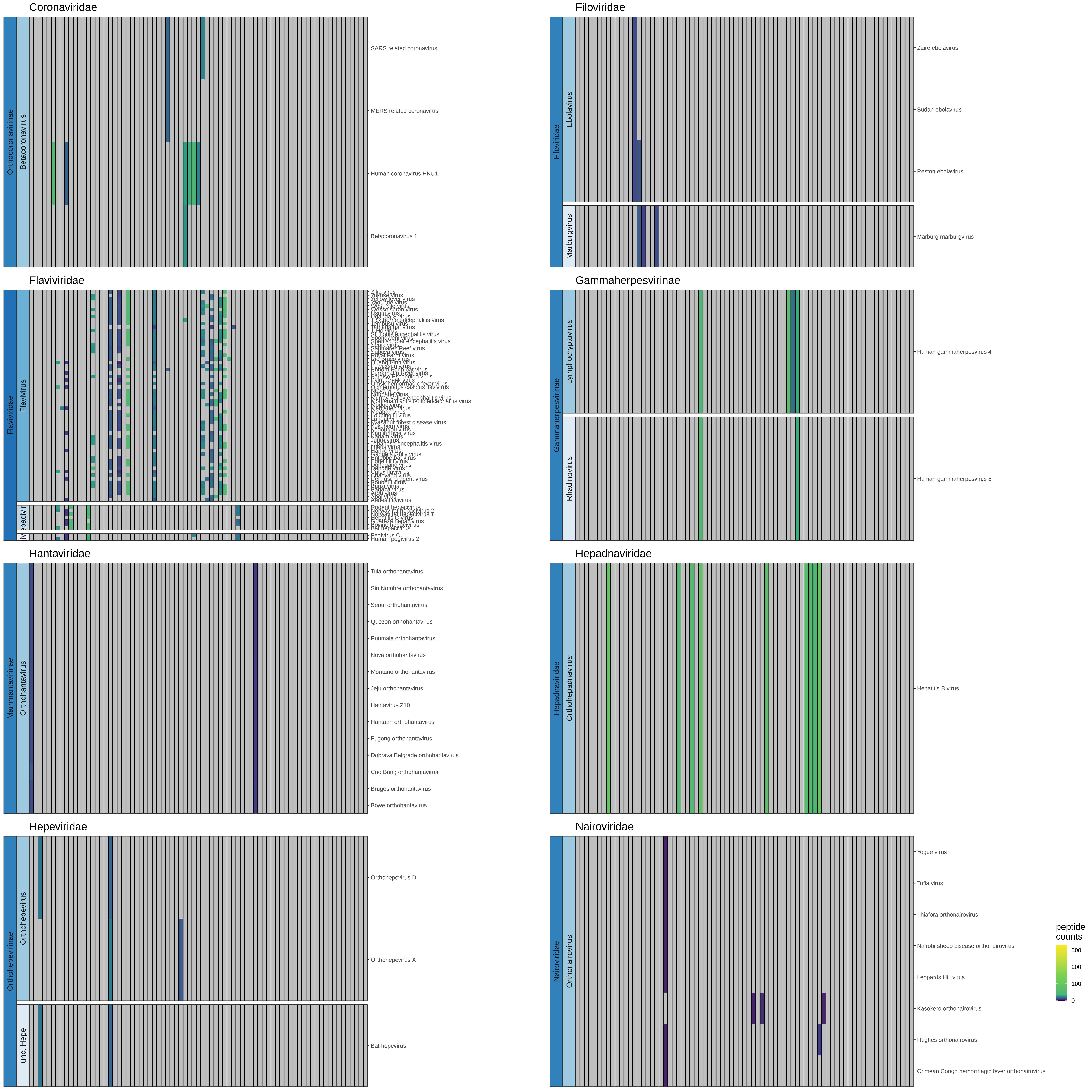

Coronaviridae
Orthocoronavirinae
Betacoronavirus
SARS related coronavirus
MERS related coronavirus
Human coronavirus HKU1
Betacoronavirus 1
Filoviridae
Filoviridae
Ebolavirus
Marburgvirus
Zaire ebolavirus
Sudan ebolavirus
Reston ebolavirus
Marburg marburgvirus
Flaviviridae
Flaviviridae
Flavivirus
Zika virus
Yokose virus
Yellow fever virus
Yaounde virus
West Nile virus
Wesselsbron virus
Usutu virus
Uganda S virus
Tick borne encephalitis virus
Tembusu virus
Tamana bat virus
T Ho virus
St Louis encephalitis virus
Spondweni virus
Spanish goat encephalitis virus
Sepik virus
Saumarez Reef virus
Saboya virus
Royal Farm virus
Rio Bravo virus
Quang Binh virus
Powassan virus
Phnom Penh bat virus
Parramatta River virus
Paraiso Escondido virus
Palm Creek virus
Omsk hemorrhagic fever virus
Ochlerotatus caspius flavivirus
Ntaya virus
Nounane virus
Murray Valley encephalitis virus
Montana myotis leukoencephalitis virus
Modoc virus
Mercadeo virus
Meaban virus
Louping ill virus
Langat virus
Kyasanur forest disease virus
Kokobera virus
Kedougou virus
Kamiti River virus
Kadam virus
Jugra virus
Japanese encephalitis virus
Ilheus virus
Hanko virus
Gadgets Gully virus
Entebbe bat virus
Edge Hill virus
Donggang virus
Dengue virus
Culex flavivirus
Chaoyang virus
Cell fusing agent virus
Bouboui virus
Banzi virus
Bagaza virus
Aroa virus
Apoi virus
Aedes flavivirus
Rodent hepacivirus
Norway rat hepacivirus 2
Norway rat hepacivirus 1
Hepatitis C virus
Guereza hepacivirus
Bovine hepacivirus
Bat hepacivirus
Pegivirus C
Human pegivirus 2
Gammaherpesvirinae
Gammaherpesvirinae
Lymphocryptovirus
Rhadinovirus
Human gammaherpesvirus 4
Human gammaherpesvirus 8
Hantaviridae
Mammantavirinae
Orthohantavirus
Tula orthohantavirus
Sin Nombre orthohantavirus
Seoul orthohantavirus
Quezon orthohantavirus
Puumala orthohantavirus
Nova orthohantavirus
Montano orthohantavirus
Jeju orthohantavirus
Hantavirus Z10
Hantaan orthohantavirus
Fugong orthohantavirus
Dobrava Belgrade orthohantavirus
Cao Bang orthohantavirus
Bruges orthohantavirus
Bowe orthohantavirus
Hepadnaviridae
Hepadnaviridae
Orthohepadnavirus
Hepatitis B virus
Hepeviridae
Orthohepevirinae
Orthohepevirus
unc. Hepe
Orthohepevirus D
Orthohepevirus A
Bat hepevirus
Nairoviridae
Nairoviridae
Orthonairovirus
Yogue virus
Tofla virus
Thiafora orthonairovirus
Nairobi sheep disease orthonairovirus
Leopards Hill virus
Kasokero orthonairovirus
Hughes orthonairovirus
Crimean Congo hemorrhagic fever orthonairovirus
peptide counts
300
200
100
0

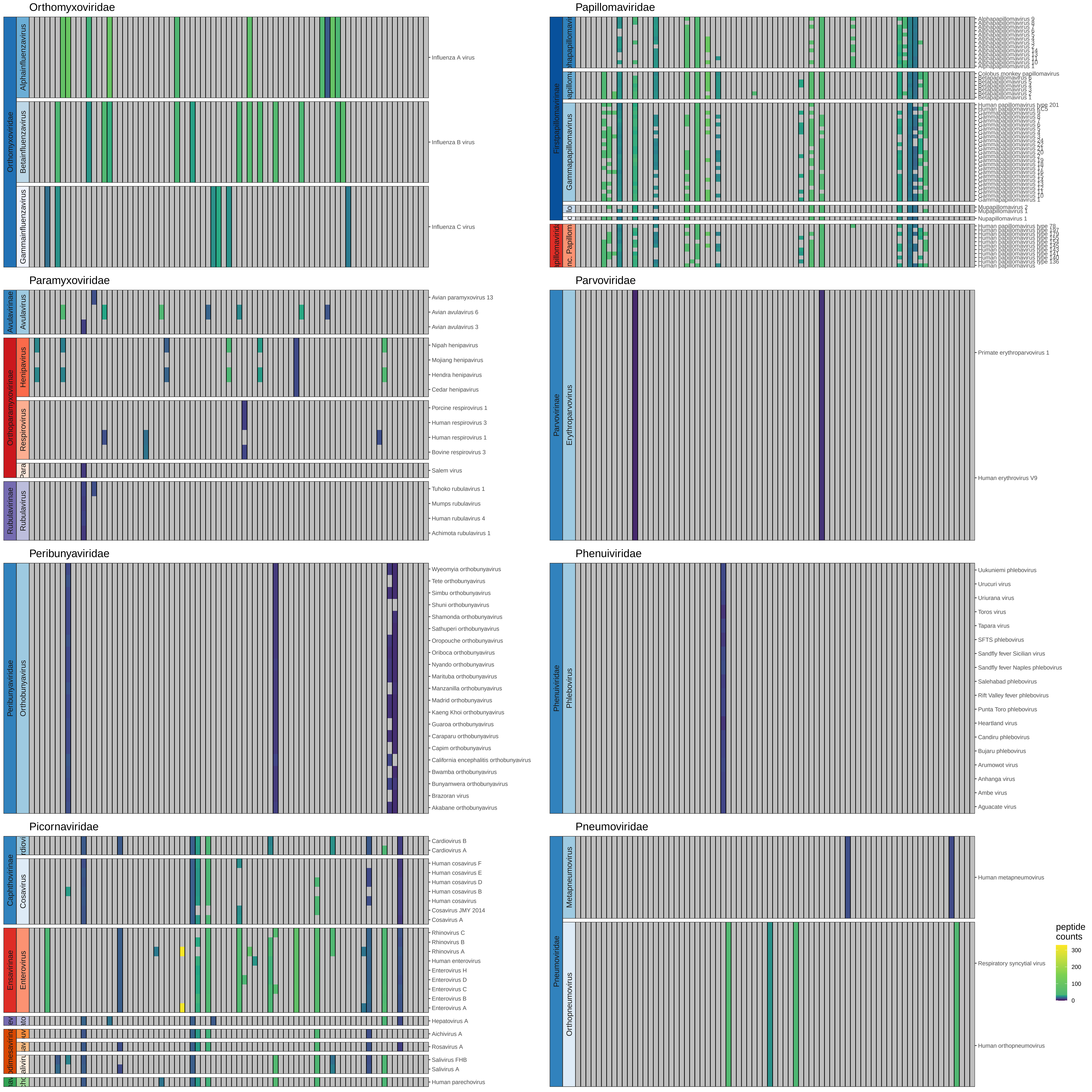
Orthomyxoviridae
Orthomyxoviridae
Alphainfluenzavirus
Betainfluenzavirus
Gammainfluenzavirus
Influenza A virus
Influenza B virus
Influenza C virus
Papillomaviridae
Firstpapillomavirinae
Gammapapillomavirus
Alphapapillomavirus 9
Alphapapillomavirus 8
Alphapapillomavirus 7
Alphapapillomavirus 6
Alphapapillomavirus 5
Alphapapillomavirus 4
Alphapapillomavirus 3
Alphapapillomavirus 2
Alphapapillomavirus 14
Alphapapillomavirus 13
Alphapapillomavirus 11
Alphapapillomavirus 10
Alphapapillomavirus 1
Colobus monkey papillomavirus
Betapapillomavirus 6
Betapapillomavirus 5
Betapapillomavirus 4
Betapapillomavirus 3
Betapapillomavirus 2
Betapapillomavirus 1
Human papillomavirus type 201
Human papillomavirus KC5
Gammapapillomavirus 9
Gammapapillomavirus 8
Gammapapillomavirus 7
Gammapapillomavirus 6
Gammapapillomavirus 5
Gammapapillomavirus 4
Gammapapillomavirus 3
Gammapapillomavirus 24
Gammapapillomavirus 22
Gammapapillomavirus 21
Gammapapillomavirus 20
Gammapapillomavirus 2
Gammapapillomavirus 19
Gammapapillomavirus 18
Gammapapillomavirus 17
Gammapapillomavirus 16
Gammapapillomavirus 15
Gammapapillomavirus 14
Gammapapillomavirus 13
Gammapapillomavirus 12
Gammapapillomavirus 11
Gammapapillomavirus 10
Gammapapillomavirus 1
Mupapillomavirus 2
Mupapillomavirus 1
Nupapillomavirus 1
Human papillomavirus type 78
Human papillomavirus type 197
Human papillomavirus type 179
Human papillomavirus type 157
Human papillomavirus type 154
Human papillomavirus type 145
Human papillomavirus type 143
Human papillomavirus type 141
Human papillomavirus type 140
Human papillomavirus type 136
Human papillomavirus
Paramyxoviridae
Avulavirinae
Avulavirus
Orthoparamyxovirinae
Henipavirus
Respirovirus
Para
Rubulavirinae
Rubulavirus
Avian paramyxovirus 13
Avian avulavirus 6
Avian avulavirus 3
Nipah henipavirus
Mojiang henipavirus
Hendra henipavirus
Cedar henipavirus
Porcine respirovirus 1
Human respirovirus 3
Human respirovirus 1
Bovine respirovirus 3
Salem virus
Tuhoko rubulavirus 1
Mumps rubulavirus
Human rubulavirus 4
Achimota rubulavirus 1
Parvoviridae
Parvovirinae
Erythroparvovirus
Primate erythroparvovirus 1
Human erythrovirus V9
Peribunyaviridae
Peribunyaviridae
Orthobunyavirus
Wyeomyia orthobunyavirus
Tete orthobunyavirus
Simbu orthobunyavirus
Shuni orthobunyavirus
Shamonda orthobunyavirus
Sathuperi orthobunyavirus
Oropouche orthobunyavirus
Oriboca orthobunyavirus
Nyando orthobunyavirus
Marituba orthobunyavirus
Manzanilla orthobunyavirus
Madrid orthobunyavirus
Kaeng Khoi orthobunyavirus
Guaroa orthobunyavirus
Caraparu orthobunyavirus
Capim orthobunyavirus
California encephalitis orthobunyavirus
Bwamba orthobunyavirus
Bunyamwera orthobunyavirus
Brazoran virus
Akabane orthobunyavirus
Phenuiviridae
Phenuiviridae
Phlebovirus
Uukuniemi phlebovirus
Urucuri virus
Uriurana virus
Toros virus
Tapara virus
SFTS phlebovirus
Sandfly fever Sicilian virus
Sandfly fever Naples phlebovirus
Salehabad phlebovirus
Rift Valley fever phlebovirus
Punta Toro phlebovirus
Heartland virus
Candiru phlebovirus
Bujaru phlebovirus
Arumowot virus
Anhanga virus
Ambe virus
Aguacate virus
Picornaviridae
Caphthovirinae
Cosavirus
Ensavirinae
Enterovirus
Cardiovirus B
Cardiovirus A
Human cosavirus F
Human cosavirus E
Human cosavirus D
Human cosavirus B
Human cosavirus
Cosavirus JMY 2014
Cosavirus A
Rhinovirus C
Rhinovirus B
Rhinovirus A
Human enterovirus
Enterovirus H
Enterovirus D
Enterovirus C
Enterovirus B
Enterovirus A
Hepatovirus A
Aichivirus A
Rosavirus A
Salivirus FHB
Salivirus A
Human parechovirus
Pneumoviridae
Pneumoviridae
Metapneumovirus
Orthopneumovirus
Human metapneumovirus
Respiratory syncytial virus
Human orthopneumovirus
peptide counts
300
200
100
0

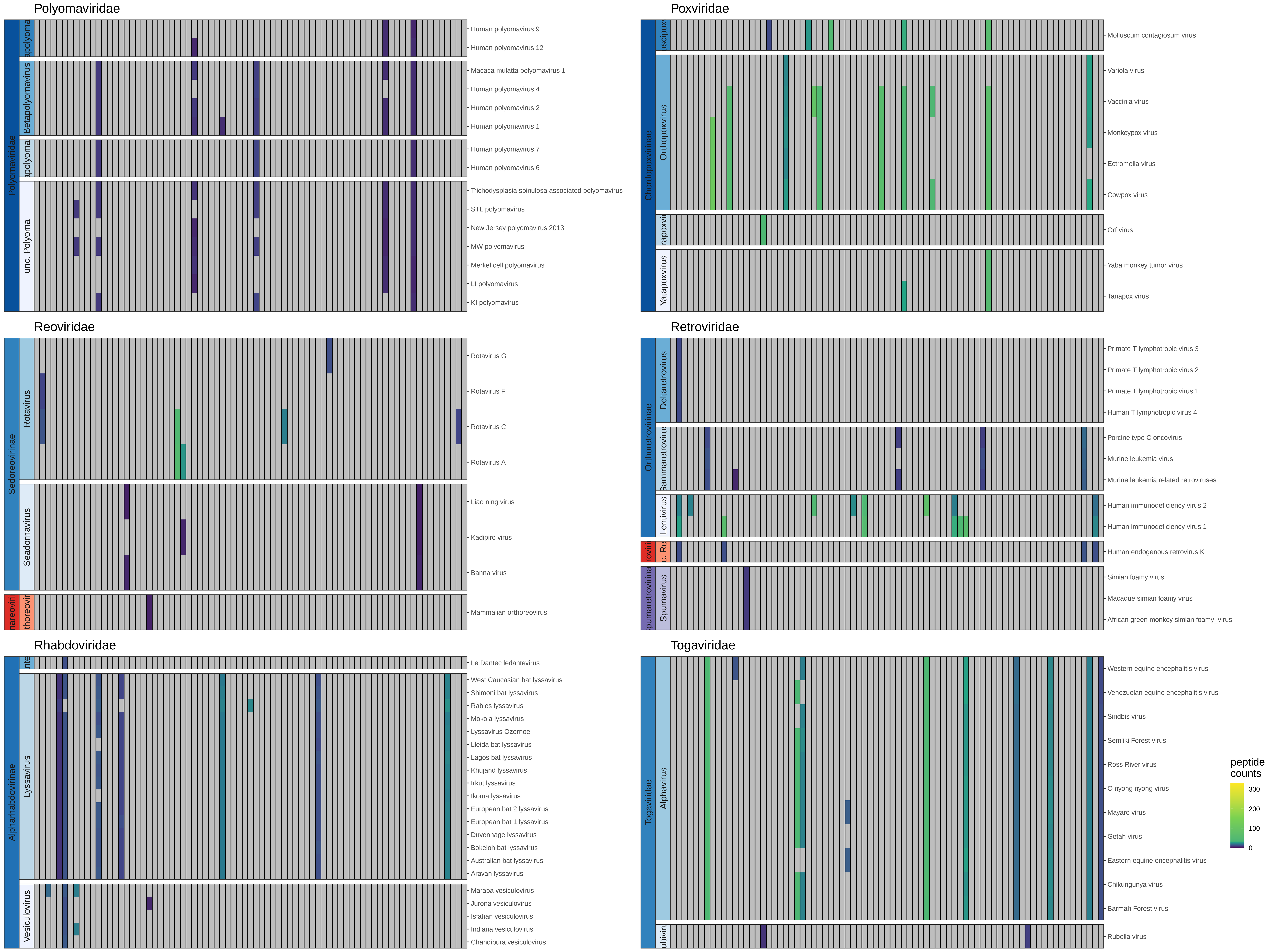

Polyomaviridae
Polyomaviridae
Betapolyomavirus
unc. Polyoma
Human polyomavirus 9
Human polyomavirus 12
Macaca mulatta polyomavirus 1
Human polyomavirus 4
Human polyomavirus 2
Human polyomavirus 1
Human polyomavirus 7
Human polyomavirus 6
Trichodysplasia spinulosa associated polyomavirus
STL polyomavirus
New Jersey polyomavirus 2013
MW polyomavirus
Merkel cell polyomavirus
LI polyomavirus
KI polyomavirus
Poxviridae
Chordopoxvirinae
Orthopoxvirus
Yatapoxvirus
Molluscum contagiosum virus
Variola virus
Vaccinia virus
Monkeypox virus
Ectromelia virus
Cowpox virus
Orf virus
Yaba monkey tumor virus
Tanapox virus
Reoviridae
Sedoreovirinae
Rotavirus
Seadornavirus
Rotavirus G
Rotavirus F
Rotavirus C
Rotavirus A
Liao ning virus
Kadipiro virus
Banna virus
Mammalian orthoreovirus
Retroviridae
Orthoretrovirinae
Deltaretrovirus
Lentivirus
Spumavirus
Primate T lymphotropic virus 3
Primate T lymphotropic virus 2
Primate T lymphotropic virus 1
Human T lymphotropic virus 4
Porcine type C oncovirus
Murine leukemia virus
Murine leukemia related retroviruses
Human immunodeficiency virus 2
Human immunodeficiency virus 1
Human endogenous retrovirus K
Simian foamy virus
Macaque simian foamy virus
African green monkey simian foamy_virus
Rhabdoviridae
Alpharhabdovirinae
Lyssavirus
Vesiculovirus
Le Dantec ledantevirus
West Caucasian bat lyssavirus
Shimoni bat lyssavirus
Rabies lyssavirus
Mokola lyssavirus
Lyssavirus Ozernoe
Lleida bat lyssavirus
Lagos bat lyssavirus
Khujand lyssavirus
Irkut lyssavirus
Ikoma lyssavirus
European bat 2 lyssavirus
European bat 1 lyssavirus
Duvenhage lyssavirus
Bokeloh bat lyssavirus
Australian bat lyssavirus
Aravan lyssavirus
Maraba vesiculovirus
Jurona vesiculovirus
Isfahan vesiculovirus
Indiana vesiculovirus
Chandipura vesiculovirus
Togaviridae
Togaviridae
Alphavirus
Western equine encephalitis virus
Venezuelan equine encephalitis virus
Sindbis virus
Semliki Forest virus
Ross River virus
O nyong nyong virus
Mayaro virus
Getah virus
Eastern equine encephalitis virus
Chikungunya virus
Barmah Forest virus
Rubella virus
peptide counts
300
200
100
0
