# Supplementary material for: Applications of VirScan to broad serological profiling of bat reservoirs for emerging zoonoses
Source: Front Public Health. 2023 Sep 22;11:1212018. doi: 10.3389/fpubh.2023.1212018 (PMC10559906; doi:10.3389/fpubh.2023.1212018)
Supplement: Supplementary file 7 [file Table_1.DOCX]

**Supplementary Information**

**Appendix 1.** Metadata from all bats – *Pteropus alecto* and *Eonycteris spelaea* – included in this analysis. Comments are from carer at the time of euthanasia.

**Table S1.**

| ID | Species | Sex | Age_cat | Age_tooth | Mass_g | Forearm_mm | Condition | Date_euthanasia | Comments |
| --- | --- | --- | --- | --- | --- | --- | --- | --- | --- |
| Pa11 | Pteropus alecto | M |  |  |  |  |  | November, 2016 |  |
| Pa12 | Pteropus alecto |  |  |  |  |  |  | November, 2016 |  |
| Pa13 | Pteropus alecto |  |  |  |  |  |  | November, 2016 |  |
| Pa14 | Pteropus alecto | M |  |  |  |  |  | November, 2016 |  |
| Pa15 | Pteropus alecto | M |  |  |  |  |  | November, 2016 |  |
| Pa16 | Pteropus alecto | M |  |  |  |  |  | November, 2016 |  |
| Pa17 | Pteropus alecto | M |  |  |  |  |  | November, 2016 |  |
| Pa18 | Pteropus alecto | M |  |  |  |  |  | November, 2016 |  |
| Pa19 | Pteropus alecto | M |  |  |  |  |  | November, 2016 |  |
| Pa20 | Pteropus alecto | M |  |  |  |  |  | November, 2016 |  |
| Pa21 | Pteropus alecto | F | Adult |  | 752 | 170 | Good | April, 2017 | White spots on liver adhesions.  Worm in liver & kidney |
| Pa23 | Pteropus alecto | F | Adult |  | 550 | 170 | Fair | April, 2017 | Significant adhesion of lung to ribs and  Diaphragm. Very early pregnant |
| Pa24 | Pteropus alecto | M | Adult |  | 625 | 170 | Fair | April, 2017 | Injured leg |
| Pa25 | Pteropus alecto | M | Sub Adult |  | 670 | 170 | Good | April, 2017 | Not sure about thymus - was in pericardium |
| Pa27 | Pteropus alecto | F | Adult |  | 670 | 165 | Good | April, 2017 | Very small fetus, slimy wings. No urine collected. Brain damaged during extraction |
| Pa30 | Pteropus alecto | M | Mature Adult |  | 640 | 170 | Fair | April, 2017 |  |
| Pa31 | Pteropus alecto | F | Mature Adult |  | 665 | 160 | Good | April, 2017 |  |
| Pa32 | Pteropus alecto | F | Adult |  | 620 | 165 | Fair | April, 2017 | Yellow flecking in liver (pathology Liver)  tiny fetus |
| Pa33 | Pteropus alecto | F | Adult |  | 665 | 165 | Good | April, 2017 | Scare on spleen & yellow flecking in liver. Worms present |
| Pa34 | Pteropus alecto | F | Adult |  | 720 | 165 | Good | April, 2017 | Yellow flecking in liver (pathology Liver) |
| Pa35 | Pteropus alecto | F | Adult |  | 615 | 165 | Good | April, 2017 | Yellow flecking in liver (pathology Liver) |
| Pa37 | Pteropus alecto | M | Juvenile | 3 | 535 | 145 | Good | October 16, 2017 | Osteomyelitis |
| Pa38 | Pteropus alecto | M | Juvenile | 1 | 400 | 140 | Good | October 16, 2017 | Damaged wing |
| Pa39 | Pteropus alecto | M | Sub Adult | 2 | 695 | 180 | Fair | October 16, 2017 | Many spots and abscess on liver |
| Pa40 | Pteropus alecto | M | Mature Adult | 7 | 715 | 175 | Poor | October 16, 2017 | Old bat. Very little body fat |
| Pa41 | Pteropus alecto | M | Adult | 5 | 660 | 160 | Fair | October 17, 2017 |  |
| Pa42 | Pteropus alecto | M | Sub Adult | 2 | 645 | 175 | Fair | October 17, 2017 | Worm in liver. Many adhesions around liver |
| Pa43 | Pteropus alecto | M | Adult | 6 | 615 | 170 | Fair | October 17, 2017 |  |
| Pa44 | Pteropus alecto | M | Adult | 6 | 750 | 170 | Good | October 18, 2017 |  |
| Pa45 | Pteropus alecto | M | Sub Adult | 4 | 650 | 175 | Good | October 18, 2017 |  |
| Pa46 | Pteropus alecto | M | Sub Adult | 2 | 620 | 170 | Fair | October 18, 2017 |  |
| Pa47 | Pteropus alecto | M | Adult | 4 | 775 | 180 | Good | October 18, 2017 | No thymus tissue |
| Pa48 | Pteropus alecto | M | Sub Adult | 2 | 670 | 170 | Good | October 18, 2017 | Dermatitus/hair thinning around body and head |
| Pa49 | Pteropus alecto | M | Sub Adult |  | 670 | 165 | Good | October 18, 2017 |  |
| Pa5 | Pteropus alecto |  |  |  |  |  |  | June, 2016 |  |
| Pa51 | Pteropus alecto | M | Adult | 6 | 835 | 175 | Good | October 19, 2017 |  |
| Pa52 | Pteropus alecto | M | Adult | 5 | 750 | 170 | Fair | October 19, 2017 | Osteomylitis |
| Pa53 | Pteropus alecto | M | Adult | 3 | 805 | 180 | Fair | October 19, 2017 | No urine |
| Pa55 | Pteropus alecto | M | Adult | 5 | 840 | 175 | Good | October 20, 2017 |  |
| Pa56 | Pteropus alecto | M | Adult | 12 | 785 | 170 | Good | October 20, 2017 |  |
| Pa57 | Pteropus alecto | M | Sub adult | 3 | 690 | 175 | Fair | October 20, 2017 |  |
| Pa58 | Pteropus alecto | F | Adult | 10 | 640 | 170 | Good | March 5, 2018 |  |
| Pa6 | Pteropus alecto |  |  |  |  |  |  | June, 2016 |  |
| Pa60 | Pteropus alecto | M | Juvenile | 1 | 530 | 175 | Poor | March 5, 2018 |  |
| Pa61 | Pteropus alecto | F | Adult | 9 | 800 | 170 | Good | March 6, 2018 | No teeth, no R wing, growth/polyp on intestine, collected mammary tissue |
| Pa62 | Pteropus alecto | M | Adult | 5 | 990 | 175 | Excellent | March 6, 2018 |  |
| Pa63 | Pteropus alecto | F | Sub Adult | 5 | 685 | 170 | Fair | March 6, 2018 |  |
| Pa64 | Pteropus alecto | F | Adult | 4 | 675 | 175 | Good | March 7, 2018 |  |
| Pa65 | Pteropus alecto | M | Adult | 7 | 815 | 160 | Excellent | March 7, 2018 |  |
| Pa66 | Pteropus alecto | F | Adult | 1 | 655 | 170 | Poor | March 7, 2018 |  |
| Pa67 | Pteropus alecto | M | Adult | 6 | 850 | 175 | Good | March 7, 2018 | No ears, large seminal vesicles |
| Pa68 | Pteropus alecto | F | Adult | 5 | 640 | 160 | Good | March 8, 2018 |  |
| Pa69 | Pteropus alecto | M | Adult | 4 | 890 | 175 | Excellent | March 8, 2018 | Small lymph nodes |
| Pa70 | Pteropus alecto | M | Adult | 5 | 905 | 170 | Excellent | March 8, 2018 | Small lymph nodes |
| Pa71 | Pteropus alecto | F | Sub Adult | 1 | 605 | 155 | Good | March 8, 2018 |  |
| Pa72 | Pteropus alecto | M | Adult | 5 | 1045 | 185 | Good | March 9, 2018 | Auxiliary small & dark, Lymph nodes smaller overall |
| Pa73 | Pteropus alecto | F | Adult | 9 | 725 | 175 | Good | March 9, 2018 | Collected mammary tissue |
| Pa74 | Pteropus alecto | M | Adult | 1 | 670 | 165 | Good | March 9, 2018 |  |
| Pa75 | Pteropus alecto | M | Adult | 3 | 870 | 175 | Good | March 12, 2018 |  |
| Pa76 | Pteropus alecto | M | Adult | 3 | 750 | 170 | Good | March 12, 2018 |  |
| Pa78 | Pteropus alecto | M | Adult | 2 | 740 | 175 | Good | March 12, 2018 |  |
| Pa79 | Pteropus alecto | F | Adult | 4 | 665 | 165 | Good | March 13, 2018 | Growth/infection under jaw, enlarged lymph nodes in neck |
| Pa8 | Pteropus alecto |  |  |  |  |  |  | June 16, 2023 |  |
| Pa80 | Pteropus alecto | F | Adult | 3 | 595 | 165 | Good | March 13, 2018 | Enlarged lymph nodes |
| Pa86 | Pteropus alecto | F | Adult |  | 580 | 165 | Poor | March 15, 2018 | Older, Scrawny, sparce hair |
| Pa87 | Pteropus alecto | M | Sub Adult |  | 555 | 155 | Fair | March 15, 2018 |  |
| Pa88 | Pteropus alecto | F | Adult |  | 605 | 160 | Good | March 15, 2018 |  |
| Pa89 | Pteropus alecto | M | Sub Adult |  | 670 | 165 | Good | September 10, 2018 |  |
| Pa9 | Pteropus alecto | M |  |  |  |  |  | November 16, 2023 |  |
| Pa90 | Pteropus alecto | M | Adult |  | 675 | 165 | Good | September 10, 2018 |  |
| Pa92 | Pteropus alecto | M | Adult |  | 610 | 160 | Good | September 10, 2018 |  |
| Pa93 | Pteropus alecto | M | Adult |  | 670 | 165 | Good | September 10, 2018 | Pregnant - well developed fetus tissues - remove for samples. Large thymus |
| Pa94 | Pteropus alecto | F | Adult |  | 755 | 165 | Good | September 11, 2018 | Rat lung worm- cysts on liver, worms encapsulated in a number of tissues and around heart |
| Pa95 | Pteropus alecto | F | Adult |  | 570 | 160 | Fair | September 11, 2018 |  |
| Pa96 | Pteropus alecto | M | Juvenile |  | 510 | 145 | Good | September 11, 2018 |  |
| Pa98 | Pteropus alecto | M | Adult |  | 735 | 175 | Good | September 11, 2018 |  |
| Pa99 | Pteropus alecto | M | Adult |  | 600 | 160 | Good | September 12, 2018 | Liver adhesions to diaphragm, spotty liver |
| Es1 | Eonycteris spelaea | F | Adult |  | 39 | 59.67 |  | October 22, 2015 |  |
| Es2 | Eonycteris spelaea | F | Adult |  | 47 | 63.47 |  | October 22, 2015 |  |
| Es4 | Eonycteris spelaea | F | Adult |  | 32 | 58.14 |  | April 13, 2016 |  |
| Es5 | Eonycteris spelaea | M | Adult |  | 94.49 | 66.33 |  | April 13, 2016 |  |
| Es6 | Eonycteris spelaea | M | Adult |  | 108.55 | 71.54 |  | April 13, 2016 |  |

**Appendix 2:** Does total body mass vary with forearm length for *P. alecto*?

We used two linear models in base R package ‘stats’ to explore how body mass (grams) covaries with forearm length (mm). We summarize the model structure and output below:

*Models:*

Model1a <- lm(Mass_g~Forearm_mm, data=subset(meta, Age_cat=="Adult" & Species=="Pteropus alecto"))

Model1b <- lm(Mass_g~Forearm_mm, data=subset(meta, Age_cat=="Sub Adult"& Species=="Pteropus alecto"))

*Output:*

**Table S2: Output from linear model for Adult and Subadult bats**

|  | Estimate | Std. error | t value | p-val |
| --- | --- | --- | --- | --- |
| *Adult bats* |  |  |  |  |
| Intercept | -1203.07 | 338.41 | -3.56 | 0.001* |
| Forearm (mm) | 11.4 | 2.0 | 5.7 | <0.0001* |
| *Subadult bats* |  |  |  |  |
| Intercept | 27.25 | 181.54 | 0.15 | 0.88 |
| Forearm (mm) | 3.68 | 1.07 | 3.43 | 0.005* |

*Statistical significance at p<0.05

**Appendix 3:** Does categorical body condition scores accurately reflect mass : forearm residuals?

We used a linear model in base R package ‘stats’ to explore whether body condition category reflects mass : forearm residuals in adult *P. alecto*. We summarize the model structure and output below:

*Model:*

Model2 <- lm(mass_residuals ~ condition, data = dat.mass)

*Output:*

**Table S3: Output from linear model for condition categories**

|  | Estimate | Std. error | t value | p-val |
| --- | --- | --- | --- | --- |
| Intercept | -85.12 | 31.83 | -2.67 | 0.01* |
| Condition - Fair | 41.41 | 34.87 | 1.18 | 0.24 |
| Condition - Good | 87.52 | 33.10 | 2.64 | 0.01* |
| Condition - Excellent | 250.00 | 42.11 | 5.94 | <0.001* |

*Statistical significance at p<0.05

**Appendix 4:** Are mass : forearm residuals related to serostatus for specific viral families?

We used a generalized linear mixed effects model in the R package ‘lme4’ to explore whether low mass : forearm residuals are associated with positive serostatus for various virus families in adult *P. alecto*. We summarize the model structure and output below:

*Model:*

Model3 <- glmer(serostatus_num~ mass_residuals:virus_genus + (1|ID), nAGQ=0, family="binomial", plot.dat)

*Output:*

**Table S4: Output from generalized linear model for serostatus**

|  | Estimate | Std. Error | z value | Pr(>\|z\|) |
| --- | --- | --- | --- | --- |
| (Intercept) | -3.38 | 0.11 | -32.17 | < 0.001* |
| mass_residuals:virus_genusAlphatorquevirus | 0.00 | 0.01 | 0.47 | 0.64 |
| mass_residuals:virus_genusAlphavirus | -0.03 | 0.00 | -8.73 | < 0.001* |
| mass_residuals:virus_genusAvulavirus | -0.01 | 0.00 | -3.58 | < 0.001* |
| mass_residuals:virus_genusBetacoronavirus | 0.02 | 0.00 | 3.63 | < 0.001* |
| mass_residuals:virus_genusBetapapillomavirus | 0.00 | 0.01 | -0.04 | 0.97 |
| mass_residuals:virus_genusBetapolyomavirus | 0.01 | 0.01 | 1.21 | 0.23 |
| mass_residuals:virus_genusCircovirus | 0.01 | 0.01 | 1.53 | 0.13 |
| mass_residuals:virus_genusDeltavirus | 0.01 | 0.00 | 2.58 | 0.01* |
| mass_residuals:virus_genusEbolavirus | 0.00 | 0.01 | 0.37 | 0.71 |
| mass_residuals:virus_genusEnterovirus | 0.02 | 0.00 | 5.08 | < 0.001* |
| mass_residuals:virus_genusErythroparvovirus | -0.01 | 0.01 | -1.57 | 0.12 |
| mass_residuals:virus_genusFlavivirus | -0.01 | 0.00 | -2.81 | 0.005* |
| mass_residuals:virus_genusGammaretrovirus | -0.02 | 0.00 | -5.25 | < 0.001* |
| mass_residuals:virus_genusHenipavirus | -0.01 | 0.00 | -2.51 | 0.01* |
| mass_residuals:virus_genusHepacivirus | 0.00 | 0.01 | 0.13 | 0.90 |
| mass_residuals:virus_genusHepatovirus | 0.00 | 0.01 | 0.07 | 0.94 |
| mass_residuals:virus_genusHusavirus | 0.00 | 0.01 | -0.61 | 0.54 |
| mass_residuals:virus_genusInfluenzavirus_A | -0.01 | 0.00 | -2.38 | 0.02* |
| mass_residuals:virus_genusInfluenzavirus_B | -0.02 | 0.00 | -5.78 | < 0.001* |
| mass_residuals:virus_genusInfluenzavirus_C | 0.01 | 0.01 | 1.95 | 0.05 |
| mass_residuals:virus_genusLentivirus | 0.00 | 0.01 | -0.85 | 0.40 |
| mass_residuals:virus_genusLymphocryptovirus | 0.00 | 0.01 | -0.15 | 0.88 |
| mass_residuals:virus_genusLyssavirus | 0.01 | 0.00 | 2.52 | 0.01* |
| mass_residuals:virus_genusMammarenavirus | 0.00 | 0.01 | -0.12 | 0.90 |
| mass_residuals:virus_genusMarburgvirus | 0.00 | 0.01 | 0.07 | 0.94 |
| mass_residuals:virus_genusMastadenovirus | -0.02 | 0.00 | -5.42 | < 0.001* |
| mass_residuals:virus_genusMetapneumovirus | 0.00 | 0.01 | 0.49 | 0.62 |
| mass_residuals:virus_genusMolluscipoxvirus | 0.00 | 0.01 | 0.08 | 0.93 |
| mass_residuals:virus_genusNorovirus | 0.01 | 0.00 | 2.99 | 0.003* |
| mass_residuals:virus_genusOrthobunyavirus | -0.01 | 0.01 | -1.08 | 0.28 |
| mass_residuals:virus_genusOrthohantavirus | 0.01 | 0.00 | 2.82 | 0.005* |
| mass_residuals:virus_genusOrthohepadnavirus | -0.01 | 0.00 | -3.00 | 0.003* |
| mass_residuals:virus_genusOrthohepevirus | 0.00 | 0.01 | -0.04 | 0.97 |
| mass_residuals:virus_genusOrthonairovirus | -0.01 | 0.00 | -2.69 | 0.01* |
| mass_residuals:virus_genusOrthopneumovirus | 0.00 | 0.01 | 0.03 | 0.97 |
| mass_residuals:virus_genusOrthopoxvirus | -0.03 | 0.00 | -7.85 | < 0.001* |
| mass_residuals:virus_genusOrthoreovirus | -0.01 | 0.01 | -1.75 | 0.08 |
| mass_residuals:virus_genusParapoxvirus | 0.01 | 0.00 | 2.45 | 0.01* |
| mass_residuals:virus_genusParechovirus | -0.01 | 0.00 | -2.94 | 0.003* |
| mass_residuals:virus_genusPegivirus | -0.01 | 0.00 | -1.99 | 0.05* |
| mass_residuals:virus_genusPhlebovirus | 0.00 | 0.01 | 0.37 | 0.71 |
| mass_residuals:virus_genusPicobirnavirus | -0.01 | 0.01 | -1.71 | 0.09 |
| mass_residuals:virus_genusRespirovirus | -0.01 | 0.01 | -1.34 | 0.18 |
| mass_residuals:virus_genusRoseolovirus | 0.00 | 0.01 | 0.37 | 0.71 |
| mass_residuals:virus_genusRotavirus | -0.02 | 0.00 | -7.02 | < 0.001* |
| mass_residuals:virus_genusRubivirus | 0.02 | 0.00 | 4.19 | < 0.001* |
| mass_residuals:virus_genusSalivirus | 0.01 | 0.01 | 1.27 | 0.20 |
| mass_residuals:virus_genusSapovirus | 0.01 | 0.01 | 0.82 | 0.41 |
| mass_residuals:virus_genusSeadornavirus | 0.01 | 0.00 | 2.79 | 0.01* |
| mass_residuals:virus_genusSimplexvirus | 0.01 | 0.01 | 1.80 | 0.07 |
| mass_residuals:virus_genusSpumavirus | 0.00 | 0.01 | 0.37 | 0.71 |
| mass_residuals:virus_genusTl2011virus | 0.01 | 0.01 | 2.12 | 0.03* |
| mass_residuals:virus_genusunc. Arena | -0.01 | 0.00 | -3.00 | 0.003* |
| mass_residuals:virus_genusunc. Cyclo | 0.00 | 0.01 | 0.37 | 0.71 |
| mass_residuals:virus_genusunc. Papilloma | 0.00 | 0.01 | 0.37 | 0.71 |
| mass_residuals:virus_genusunc. Polyoma | 0.00 | 0.01 | 0.37 | 0.71 |
| mass_residuals:virus_genusVesiculovirus | -0.01 | 0.01 | -0.97 | 0.33 |

*Statistical significance at p<0.05

**Appendix 5:**

We used two generalized linear models in base R package ‘stats’ to explore whether mass : forearm residuals or age are predictors of total peptide hits and/or total viral exposures in adult *P. alecto*. We summarize the model structure and output below:

*Model:*

Model4a <- glm(tot_hits~mass_residuals + age_tooth, data = ind.dat, family="poisson")

Model4b <- glm(N_exposures~mass_residuals + age_tooth, data = ind.dat, family="poisson")

*Output:*

**Table S5: Output from linear model**

|  | Estimate | Std. error | z value | Pr(>\|z\|) |
| --- | --- | --- | --- | --- |
| *Total peptide hits* |  |  |  |  |
| Intercept | 5.99 | 0.016 | 364.63 | <0.0001* |
| Mass_residuals | -0.001 | 0.0001 | -7.65 | <0.0001* |
| Age_tooth | 0.024 | 0.003 | 7.96 | <0.0001* |
| *Total viral exposures* |  |  |  |  |
| Intercept | 2.14 | 0.107 | 20.02 | <0.0001* |
| Mass_residuals | -0.004 | 0.001 | -5.57 | <0.0001* |
| Age_tooth | 0.04 | 0.019 | 2.11 | 0.04 |
